# PHF6 suppresses self-renewal of leukemic stem cells in AML

**DOI:** 10.1038/s41375-024-02340-5

**Published:** 2024-07-14

**Authors:** Sapana S. Jalnapurkar, Aishwarya S. Pawar, Subin S. George, Charles Antony, Patrick Somers, Jason Grana, Victoria K. Feist, Sandeep Gurbuxani, Vikram R. Paralkar

**Affiliations:** 1 Division of Hematology and Oncology, Department of Medicine, University of Pennsylvania Perelman School of Medicine, Philadelphia, PA, USA.; 2 Biomedical Graduate Studies, University of Pennsylvania Perelman School of Medicine, Philadelphia, PA, USA.; 3 Institute for Biomedical Informatics, University of Pennsylvania Perelman School of Medicine, Philadelphia, PA, USA.; 4 Department of Pathology, University of Chicago, Chicago, IL, USA.; 5 Department of Cell and Developmental Biology, University of Pennsylvania Perelman School of Medicine, Philadelphia, PA, USA.; 6 Abramson Family Cancer Research Institute, University of Pennsylvania Perelman School of Medicine, Philadelphia, PA, USA.; 7 These authors contributed equally: Sapana S. Jalnapurkar, Aishwarya S. Pawar.

## Abstract

Acute myeloid leukemia is characterized by uncontrolled proliferation of self-renewing myeloid progenitors accompanied by a differentiation arrest. PHF6 is a chromatin-binding protein mutated in myeloid leukemias, and its isolated loss increases mouse HSC self-renewal without malignant transformation. We report here that *Phf6* knockout increases the aggressiveness of *Hoxa9*-driven AML over serial transplantation, and increases the frequency of leukemia initiating cells. We define the in vivo hierarchy of *Hoxa9*-driven AML and identify a population that we term the “LIC-e” (leukemia initiating cells enriched) population. We find that *Phf6* loss expands the LIC-e population and skews its transcriptome to a more stem-like state; concordant transcriptome shifts are also observed on *PHF6* knockout in a human AML cell line and in *PHF6* mutant patient samples from the BEAT AML dataset. We demonstrate that LIC-e accumulation in *Phf6* knockout AML occurs not due to effects on cell cycle or apoptosis, but due to an increase in the fraction of its progeny that retain LIC-e identity. Our work indicates that *Phf6* loss increases AML self-renewal through context-specific effects on leukemia stem cells.

## INTRODUCTION

*PHF6* (Plant homeodomain-like finger protein 6) is an X-chromosome gene mutated in a variety of myeloid and lymphoid leukemias. PHF6 localizes to the nucleus and is known to interact with chromatin, but its precise molecular function is poorly understood, with reported roles ranging from cell cycle control [1–3], DNA repair [3, 4], to transcriptional regulation [5–8]. Somatic *PHF6* mutations are seen in 38% of T-cell acute lymphocytic leukemia (T-ALL) [9], in 3–6% of AML, myelodysplastic syndrome (MDS), and chronic myelomonocytic leukemia (CMML), and in 23% of mixed-phenotype acute leukemia (MPAL) and undifferentiated leukemia [10–16]. *PHF6* mutations co-occur in MDS/AML with mutations in *RUNX1, ASXL1*, and *U2AF1* [11, 13, 16], with the majority of *PHF6* mutations being frameshift and nonsense mutations distributed throughout the gene body [16], predicted to produce null alleles and indicating that *PHF6* acts as a leukemia suppressor.

Germline *Phf6* deletion in mice leads to perinatal lethality, while mice with hematopoietic *Phf6* deletion are viable and fertile [17, 18]. Conditional hematopoietic knockouts using multiple *Cre* systems have consistently shown minimal alterations to homeostatic hematopoiesis, but striking increases in HSC self-renewal on transplantation, with the ability to engraft beyond five serial transplants without exhaustion, malignant transformation, or lineage skewing [17–19]. *Phf6* knockout HSCs from aged mice show transcriptional profiles similar to young HSCs, and deletion of *Phf6* from older mice shows a shift towards a younger HSC transcriptome [4]. Combination of *Phf6* loss with overexpression of activating mutants of *Notch1* [18] or *Jak3* [20], or overexpression of wildtype *Tlx3* [17] has been shown to cause T-ALL acceleration, while transgenic crosses of *Phf6* deletion with *Idh2* mutation produce mixed myeloid-lymphoid leukemias [21]. Collectively, the role of PHF6 appears to be the repression of self-renewal, both in normal HSCs as well as in T-ALL [18]. However, there are also reports of PHF6 being required for the growth of B-ALL [22], and more recently, for the growth of AML driven by *BCR-ABL, AML1-ETO*, or *MLL-AF9* fusions [23]. The latter publication reporting the counterintuitive finding that *Phf6* loss reduces AML growth and stemness contradicts the model of PHF6 as a leukemia suppressor; however, the publication’s use of fusion protein drivers that do not co-occur with human *PHF6* mutations may indicate that the chosen AML models recapitulated narrow disease subsets potentially not reflective of broader AML biology. The precise role of *Phf6* loss in AML therefore remains unclear.

In this study, we use *Hoxa9* retroviral transduction as a model of mouse AML [24] that is broadly relevant, given that >70% of human AMLs overexpress *HOXA9* [25]. We examine the role of hematopoietic *Phf6* deletion on AML progression and show that *Phf6* loss accelerates AML progression over serial transplantation, accompanied by an accumulation of leukemia initiating cells (LICs). We identify that LICs in the *Hoxa9* transduction model are concentrated within a small Kit+ Ly6C− subpopulation that we term “LIC-e” (LIC-enriched). We also show that, contrary to prior reports, *Phf6* loss has no effect on cell cycle or apoptosis, but instead increases the fraction of LIC-e progeny that retain persistent LIC-e identity. We further show that *Phf6* loss leads LIC-e cells to gain a more stem-like transcriptome, with reduced accessibility of genomic regions bound by the transcription factors AP-1 family, GATA2, and SPI1, among others. *PHF6* knockout in the human THP-1 AML cell line, as well as a comparison of *PHF6* mutated and unmutated samples from the BEAT AML dataset [26], similarly show a transcriptome shift towards stemness. Collectively, our data resolves a controversy in the literature by demonstrating that PHF6 suppresses AML stem cell self-renewal in a clinically relevant AML model system, and demonstrates how the loss of a specific repressor of HSC self-renewal drives leukemia stemness.

## RESULTS

### PHF6 loss increases leukemia initiating cell frequency in *Hoxa9*-driven AML

**Fig. 1:**
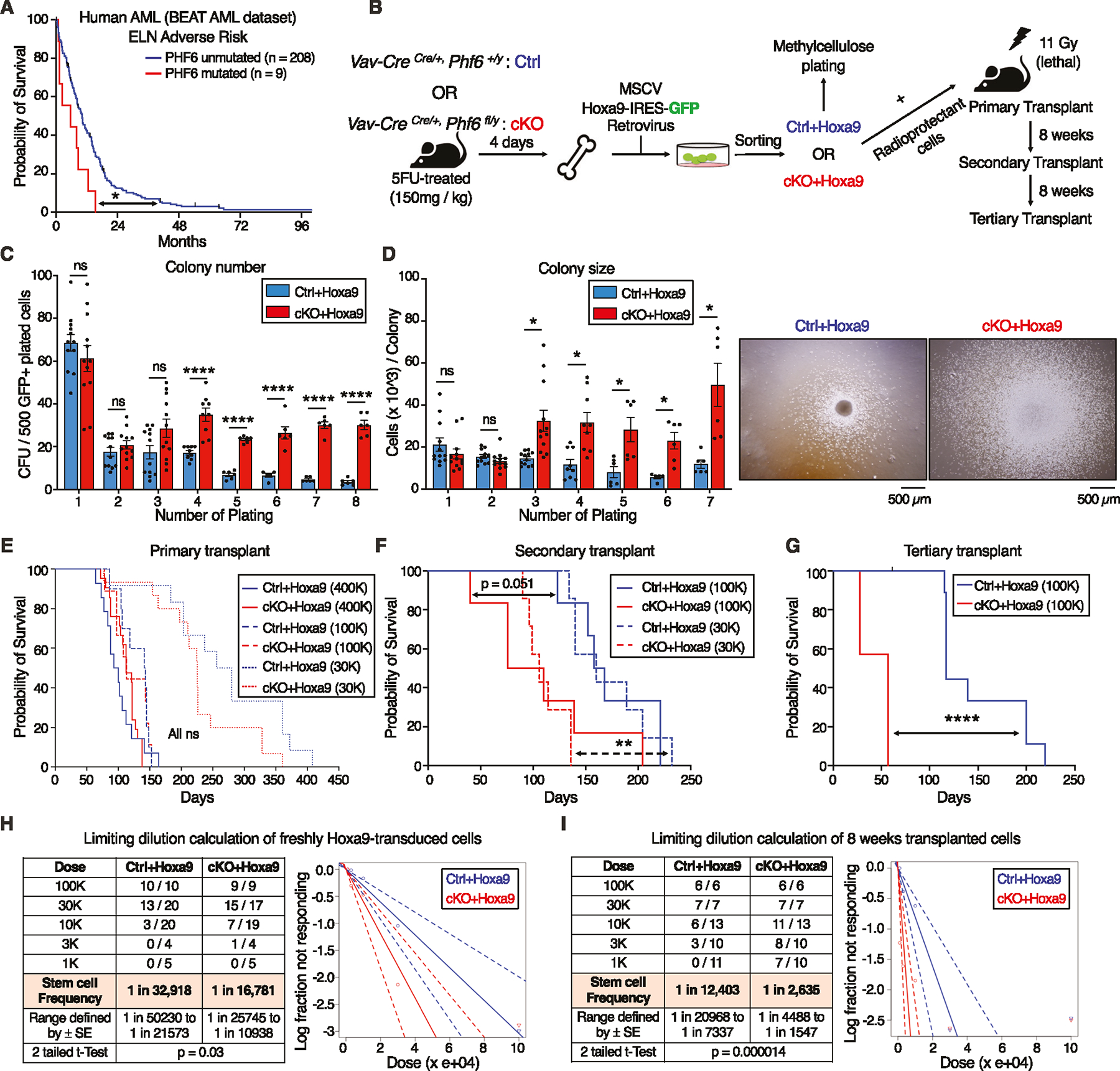
*Phf6* loss increases leukemia initiating cell frequency in *Hoxa9*-driven AML. **A** Kaplan-Meier survival curve for *PHF6* mutated and unmutated adverse risk adult AML patients (ELN classification) from the BEAT AML dataset. **B** Experimental design for *Hoxa9* retroviral transduction of *Ctrl* and *cKO* marrow, followed by colony forming unit assay (CFU), AML induction in mice, and serial transplantation of transformed leukemic cells. **C** Bar graph showing number of colony forming units (CFUs) obtained from 8 rounds of serial methylcellulose replating of 500 cells/plate of *Ctrl*+*Hoxa9* and *cKO*+*Hoxa9* transformed mouse bone marrow. (n = 6–12 biological replicates). **D** *Left* Bar graph showing average number of cells per colony (colony size) obtained after 7 rounds of serial methylcellulose replating of 500 cells/plate of *Ctrl*+*Hoxa9* and *cKO*+*Hoxa9* transformed mouse bone marrow. (n = 6–12 biological replicates) *Right* Representative photographs of colonies at the 3rd plating. Scale bar represents 500 μm. **E** Kaplan–Meier survival curves of *Ctrl*+*Hoxa9* and *cKO*+*Hoxa9* primary transplant recipients receiving 400 K, 100 K, or 30 K GFP+ cells. (n = 7–21 mice per cohort). **F** Kaplan–Meier survival curve of *Ctrl*+*Hoxa9* and *cKO*+*Hoxa9* secondary transplant recipients, receiving 100 K or 30 K GFP+ cells harvested from bone marrow of primary recipients 8 weeks after transplantation. (n = 7–10 mice per cohort). **G** Kaplan–Meier survival curve of *Ctrl*+*Hoxa9* and *cKO*+*Hoxa9* tertiary transplant recipients, receiving 100 K GFP+ cells harvested from bone marrow of secondary recipients 8 weeks after transplantation. (n = 7–11 mice per cohort). **H**, **I** Limiting dilution analysis for LIC calculation for (**H**) freshly *Hoxa9*-transduced cells, (**I**) 8 weeks primary transplanted leukemic cells. All bar graphs show mean ± SEM and statistical significance was calculated using the Student t-test. For all survival curves, statistical significance was calculated using the Log-rank (Mantel-Cox) test. *p < 0.05, **p < 0.01, ***p < 0.001; ****p < 0.0001, *ns* = *not significant*.

To determine the prognostic significance of *PHF6* mutations in human AML, we used publicly available mutational and survival data from the BEAT AML dataset [26]. Of 805 AML patients, 22 (2.7%) had *PHF6* mutations. *PHF6* mutations were associated with reduced overall survival in adverse risk patients (Fig. 1A) *PHF6* gene mutational classes (frameshift, nonsense, missense) showed similar poor survival curves relative to each other (Fig. S1A–C).

To assess the role of PHF6 loss in mouse AML, we generated conditional hematopoietic *Phf6* knockout *Vav-Cre*^*Cre*/+^*Phf6*^*fl/y*^ (*cKO*) mice (Fig. S1D) and compared them to their *Vav-Cre*^*Cre*/+^*Phf6*^+/*y*^ (*Ctrl*) littermates. Published studies of hematopoietic *Phf6* loss have reported no evidence of leukemic transformation [17–19, 27]. We also found no blood count abnormalities in *cKO* mice up to 9 months (Fig. S1E–K), further indicating that *Phf6* loss alone is likely insufficient to initiate leukemia.

We next induced AML using the *Hoxa9* retroviral transduction model. We picked this model due to its ability to produce AML with a relatively longer latency (lethality in ~3–6 months) [24], allowing us to test a potential role for *Phf6* loss in accelerating AML kinetics. We transduced whole bone marrow from 5-FU-treated *Ctrl* and *cKO* mice with *MSCV Hoxa9-IRES-GFP* retrovirus, and investigated the effect of *Phf6* loss on the ability of *Hoxa9*-transformed cells to form colonies in methylcellulose (Fig. 1B). We observed that *Ctrl+Hoxa9* cells were nearly exhausted after 4 platings, whereas *cKO+Hoxa9* cells demonstrated persistent colony-forming ability up to 8 platings (Fig. 1C), with larger colonies (Fig. 1D). Thus, *Phf6* loss gives a replating advantage to *Hoxa9*-transformed marrow and delays its in vitro exhaustion. Conversely, *MLL-AF9-IRES-GFP* transformed marrow showed no evidence of exhaustion with replating, and no evidence of further acceleration with *Phf6* loss (Fig. S2A, B). Given that 70% of human AMLs show high *HOXA9* levels [25], we deemed the *Hoxa9* overexpression mouse model as being broadly relevant to human leukemia biology, as opposed to *MLL-AF9*, which does not co-occur with *PHF6* mutation in humans. We therefore proceeded with the *Hoxa9*-driven model for our studies.

To test the role of *Phf6* loss in the development of AML in vivo, we transplanted *Hoxa9*-transduced marrow into lethally irradiated syngeneic recipients (Fig. 1B). We confirmed that *Ctrl*+*Hoxa9* marrow produced lethality in recipient mice in ~3–5 months after transplantation (Fig. 1E) due to AML characterized by >20% blasts in the marrow (Fig. S2C), peripheral leukocytosis (Fig. S2D), splenomegaly with effacement of splenic architecture (Fig. S2E), and infiltration of leukemic cells in the liver (Fig. S2F). Survival was similar between *Ctrl*+*Hoxa9* and *cKO*+*Hoxa9* groups in primary recipients transplanted with multiple doses (400 K, 100 K, or 30 K cells) (Fig. 1E), with similar degrees of leukemic infiltrate (Fig. S3A–F) and splenomegaly at morbidity (Fig. S3G). However, secondary and tertiary transplantation of marrow showed progressively accelerated lethality in *cKO*+*Hoxa9* compared to *Ctrl*+*Hoxa9* (Fig. 1F, G, S3H). Thus, *Phf6* loss accelerates *Hoxa9*-driven mouse AML on serial transplantation.

We next sought to determine the effect of *Phf6* loss on the frequency of leukemia initiating cells (LIC), the sub-population of transformed marrow capable of initiating leukemia. We performed limiting dilution transplantation assays (LD) on freshly transduced marrow (GFP+ cells sorted 2 days after retroviral transduction) as well as on marrow from recipients (GFP+ bone marrow cells sorted from recipients 8 weeks after primary transplantation). We picked the 8-week time point based on the initiation of lethality in this model at ~12 weeks (Fig. 1E). LD of freshly transduced marrow showed that, at baseline, *cKO*+*Hoxa9* cells had a 2-fold higher frequency of cells capable of leukemic transformation (Fig. 1H). LD on marrow extracted 8 weeks post-transplantation showed an approximately 5-fold greater frequency of leukemia initiating cells (LICs) in *cKO*+*Hoxa9* marrow (Fig. 1I). Thus, *Phf6* loss increases LIC frequency in *Hoxa9*-driven AML, with the increase occurring during in vivo AML evolution.

### *Phf6* loss increases leukemic disease burden

**Fig. 2:**
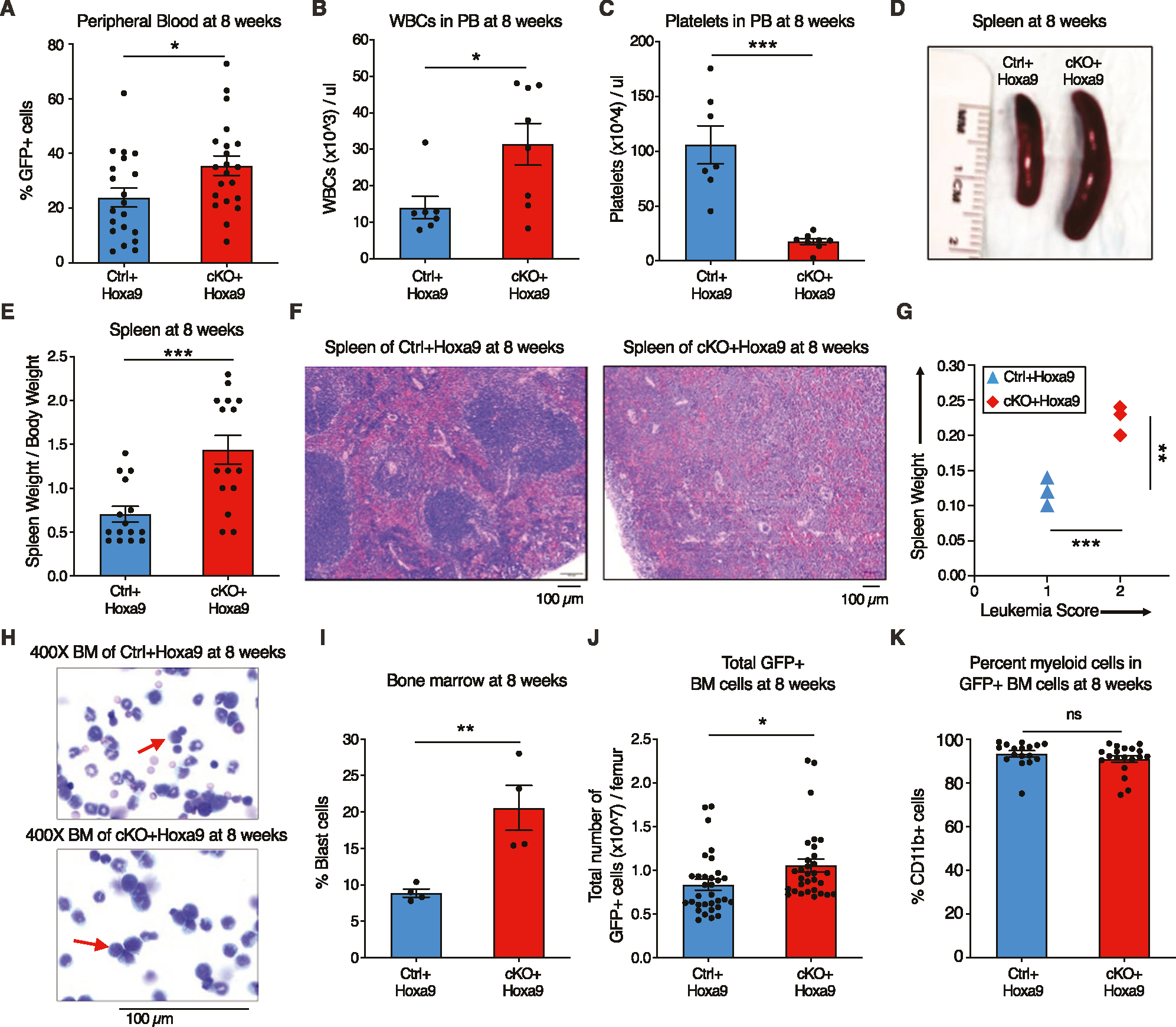
*Phf6* loss increases leukemic disease burden. **A–C** Bar graphs showing peripheral blood analysis at 8 weeks after transplantation of *Ctrl*+*Hoxa9* and *cKO*+*Hoxa9* cells. **A** Percentage of GFP+ cells in peripheral blood. **B**, **C** Counts of (**B**) WBCs and (**C**) platelets in peripheral blood. Normal range for WBCs: 2000–10,000/μl. Normal range for platelets: 900–1600 × 10^3^/μl [41]. **D** Representative photograph of spleens at 8 weeks after transplantation. Ruler depicts length in centimeters. **E** Bar graph showing spleen weight (expressed as percentage of total body weight) of primary recipients at 8 weeks after transplantation of *Ctrl*+*Hoxa9* and *cKO*+*Hoxa9* cells. **F** Representative image of H&E staining of spleen from *Ctrl*+*Hoxa9* and *cKO*+*Hoxa9* at 8 weeks after transplantation. Scale bar is 100 μm at 10X. **G** Spleen weight (y-axis) and leukemia score (x-axis) from *Ctrl*+*Hoxa9* and *cKO*+*Hoxa9* primary recipients at 8 weeks. X-axis represents a previously published leukemia infiltration score [28] calculated based on splenic architecture. Intact white and red pulp was scored as 0, extramedullary hematopoiesis evident by aberrant cells in disturbed white pulp was scored as 1, while infiltration with leukemic blasts with high mitotic activity was scored as 2. **H** Representative image of Wright–Giemsa staining of cytospin of *Ctrl*+*Hoxa9* and *cKO*+*Hoxa9* bone marrow cells at 8 weeks after transplantation. Scale bar is 100 μM at 400X. Red arrows indicate representative blast cells. **I** Bar graph of percentage blast cells of total nucleated cells in bone marrow at 8 weeks after transplant. **J**, **K** Bar graphs showing (**J**) absolute number of GFP+ cells in marrow and (**K**) percentage of CD11b+ myeloid cells among all GFP+ cells at 8 weeks after transplantation. All bar graphs show mean ± SEM and statistical significance was calculated using the Student t-test. For all survival curves, statistical significance was calculated using the Log-rank (Mantel-Cox) test. *p < 0.05, **p < 0.01, ***p < 0.001; ****p < 0.0001, *ns* = *not significant*.

To characterize the effect of *Phf6* loss further, we analyzed peripheral blood, splenic architecture, and bone marrow leukemic cell burden of primary recipients at 8 weeks after transplantation. Mice transplanted with *cKO*+*Hoxa9* cells showed a higher frequency of GFP+ cells in peripheral blood at 8 weeks than mice receiving *Ctrl*+*Hoxa9* cells (Fig. 2A). The *cKO*+*Hoxa9* group also had greater leukocytosis (Fig. 2B) and more severe thrombocytopenia (Fig. 2C). Mice in both groups displayed comparable levels of anemia (Fig. S4A, B). The *cKO*+*Hoxa9* group had increased spleen size and weight compared to the *Ctrl*+*Hoxa9* group (Fig. 2D, E), and histopathological analysis showed greater effacement of splenic architecture (Fig. 2F). Splenic infiltration was quantified using a previously described leukemia infiltration score [28], and was found to be greater in *cKO*+*Hoxa9* mice compared to *Ctrl*+*Hoxa9* (Fig. 2G). Giemsa-stained cytospin preparations showed higher blast percentages in *cKO*+*Hoxa9* at the 8-week timepoint (Fig. 2H, I), and flow cytometry showed higher absolute and percent GFP+ cells (Fig. 2J, S4C). All GFP+ cells were myeloid for both groups (Fig. 2K, S4D). Thus, while mice from both groups succumbed at similar times after primary transplant (Fig. 1F), analyses at matched time points before the onset of mortality revealed greater disease burden in *cKO*+*Hoxa9* mice compared to *Ctrl*+*Hoxa9*.

### *Phf6* loss increases the frequency of self-renewing, transplantable LICs

**Fig. 3:**
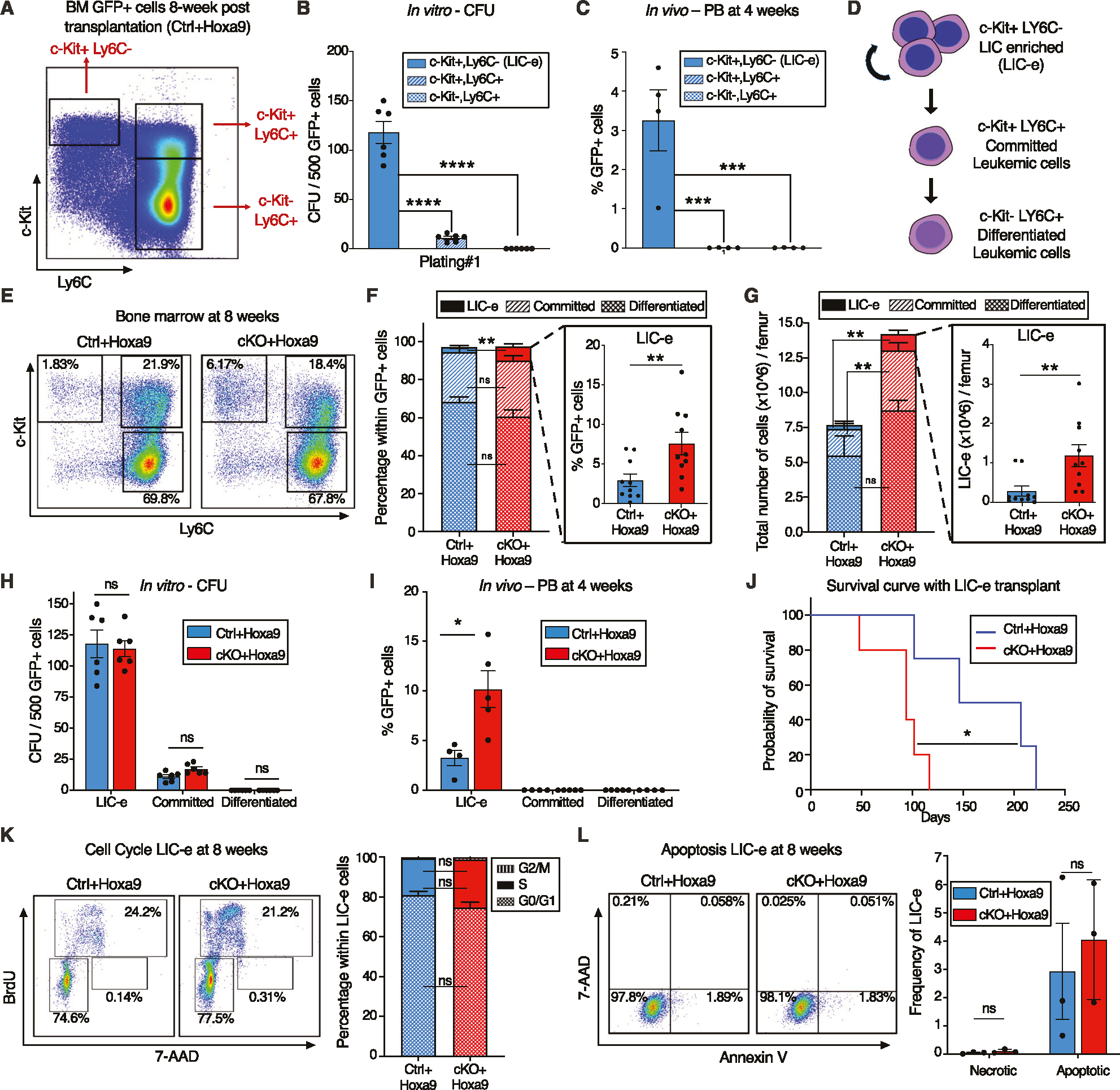
*Phf6* loss increases the frequency of self-renewing, transplantable LICs. **A** Representative flow cytometry plot of bone marrow GFP+ cells at 8 weeks after transplantation with *Ctrl*+*Hoxa9* cells, compartmentalized into three subpopulations (i) LIC-e, (ii) Committed, and (iii) Differentiated leukemic cells using c-Kit and Ly6C expression. Note: The same flow cytometry plot has been shown in Fig. S5 with detailed immunophenotypic markers. **B** Bar graph showing number of colony forming units (CFUs) obtained on methylcellulose plating of 500 cells of sorted subpopulations of *Ctrl*+*Hoxa9* transplanted marrow. (n = 6 biological replicates). **C** Bar graph showing frequencies of GFP+ cells in peripheral blood of secondary recipient mice at 4 weeks after transplantation of sorted subpopulations from *Ctrl*+*Hoxa9* primary recipient marrow. (n = 4–5 biological replicates). **D** Schematic of hierarchical organization of leukemic cells (LIC-e, committed, and differentiated leukemic cells) within AML produced by retroviral *Hoxa9* transduction. **E** Representative flow cytometry plots depicting subpopulations of *Ctrl*+*Hoxa9* and *cKO*+*Hoxa9* leukemia marrow at 8 weeks after primary transplant. **F**, **G** Stacked bar graphs showing (**F**) frequencies, and (**G**) absolute number per femur of LIC-e, committed, and differentiated leukemic populations from *Ctrl*+*Hoxa9* and *cKO*+*Hoxa9* marrow at 8 weeks after transplantation. Insets show frequencies and absolute numbers of the LIC-e subpopulation. (n = 10–11). **H** Bar graph showing number of CFUs obtained on methylcellulose plating of 500 cells of sorted LIC-e, committed, and differentiated leukemic populations from *Ctrl*+*Hoxa9* and *cKO*+*Hoxa9* primary recipient bone marrow at 8 weeks after transplantation. (n = 6 biological replicates). **I** Bar graph showing frequencies of GFP+ cells in the peripheral blood of secondary recipient mice at 4 weeks after transplantation with sorted LIC-e, committed, and differentiated leukemic cell subpopulations from *Ctrl*+*Hoxa9* and *cKO*+*Hoxa9* primary recipient bone marrow at 8 weeks after transplantation. (n = 5 biological replicates). Note: The *Ctrl*+*Hoxa9* samples in (**H**) and (**I**) are the same as those depicted in (**B**) and (**C**)—the current figures are comparing *Ctrl* and *cKO*. **J** Kaplan–Meier curve of secondary transplant recipients receiving 50 K sorted LIC-e cells from *Ctrl*+*Hoxa9* and *cKO*+*Hoxa9* primary mouse bone marrow at 8 weeks after transplantation. (n = 4–5 biological replicates). **K** *Left* Representative flow cytometry plots for cell cycle analysis of LIC-e cells, with BrdU (marking cells in S phase) and 7-AAD (marking DNA) 2 h after BrdU injection into live mice at 8 weeks after transplantation. *Right* Stacked bar graph indicates frequencies of in vivo LIC-e cells in G0/G1, S, and G2/M phases. (n = 13 biological replicates). **L** *Left* Representative flow cytometry plots of apoptotic (Annexin V+, 7AAD−), and necrotic (7AAD+) LIC-e cells. *Right* Bar graph shows frequencies of apoptotic and necrotic LIC-e cells from *Ctrl*+*Hoxa9* and *cKO*+*Hoxa9* primary recipient bone marrow at 8 weeks after transplantation. All bar graphs show mean ± SEM and statistical significance was calculated using the Student t-test. For all survival curves, statistical significance was calculated using the Log-rank (Mantel-Cox) test. *p < 0.05, **p < 0.01, ***p < 0.001; ****p < 0.0001, *ns* = *non significant*.

To characterize the immunophenotype of AML subpopulations (including LICs), we further analyzed the marrow of *Ctrl*+*Hoxa9* recipients at 8 weeks after transplantation. The GFP+ cells did not express B or T cell markers (Fig. S5A). Immature AML cells are known to have high c-Kit expression [29], and leukemic stem cells (LSCs) in the *MLL-AF9* retroviral mouse model aberrantly express mature myeloid lineage antigens such as Ly6C and CD11b [30]. To identify the corresponding subpopulation containing LICs in *Hoxa9*-only-driven AML, and to characterize the differentiation hierarchy of this model, we settled on a strategy using c-Kit and Ly6C expression to divide GFP+ marrow cells into three populations: (i) cKit+ Ly6C−, (ii) c-Kit+ Ly6C+, and (iii) c-Kit− Ly6C+ (Fig. 3A, S5B). The population at the top of the hierarchy was the cKit+ Ly6C− population, an immature population with expression of cKit, CD34, and dim CD11b, with no expression of Ly6C, Ly6G, or Sca-1, and mixed expression of CD16/32 (Fig. S5C). This population was capable of giving rise to more differentiated Ly6C+ cells within 2 days of culture (Fig. S5D), could produce colonies on methylcellulose plating (Fig. 3B), and could engraft into recipient mice (Fig. 3C). Based on this subpopulation’s ability to engraft, but cognizant that not all cells within it are LICs, we termed it the ‘LIC enriched’ (LIC-e) population (Fig. 3D). The second population was the c-Kit+ Ly6C + population, also expressing CD11b, CD34, and CD16/32, but not Sca-1 (Fig. S5C). On culture, this population could only give rise to Ly6C+ cells, but not to any Ly6C- cells (Fig. S5D), indicating that it is irreversibly committed to differentiation. This population could produce a small number of colonies in methylcellulose (Fig. 3B), but could not engraft mice (Fig. 3C). We termed it the ‘committed’ leukemic cell population (Fig. 3D). The third population was the c-Kit− Ly6C+ population, which expressed Ly6C, CD11b, CD34, and CD16/32, but not Sca-1, and had mixed expression of Ly6G (Fig. S5C). It could not produce any colonies (Fig. 3B) nor engraft (Fig. 3C). In vitro, this population only gave rise to Ly6C+ cells (Fig. S5D). We termed it the “differentiated” leukemic cell population (Fig. 3D). We thus established the hierarchical organization of *Hoxa9*-driven AML, and used it to evaluate the effects of *Phf6* loss.

The LIC-e population, though comprising a small minority of GFP+ cells, was expanded in 8-week *cKO*+*Hoxa9* marrow compared to *Ctrl*+*Hoxa9*, while the relative fractions of committed and differentiated leukemic cells were similar (Fig. 3E, F). The difference in the LIC-e population was even more pronounced when absolute cell numbers were considered, showing a 5-fold increase in *cKO*+*Hoxa9* (Fig. 3G). To determine functional differences between *Ctrl*+*Hoxa9* and *cKO*+*Hoxa9* subpopulations, we sorted equal numbers of cells from each subpopulation at 8 weeks after transplant and performed methylcellulose culture and secondary transplantation into irradiated recipients. Committed and differentiated leukemic cells of either group formed few to no colonies, while LIC-e cells showed comparable colony-forming ability (Fig. 3H). *cKO*+*Hoxa9* LIC-e cells showed greater engraftment in secondary recipients at 4 weeks (Fig. 3I) and led to more rapid lethality than *Ctrl*+*Hoxa9* LIC-e cells (Fig. 3J). We did not observe any difference in cell cycle distribution or apoptosis of LIC-e cells (Fig. 3K, L). Thus, *Phf6* loss led to an expanded and more transplantable LIC-enriched AML subpopulation whose enhanced leukemic potential was not explained by differences in cell cycle or apoptosis.

### *Phf6* loss promotes a stemness gene network

**Fig. 4:**
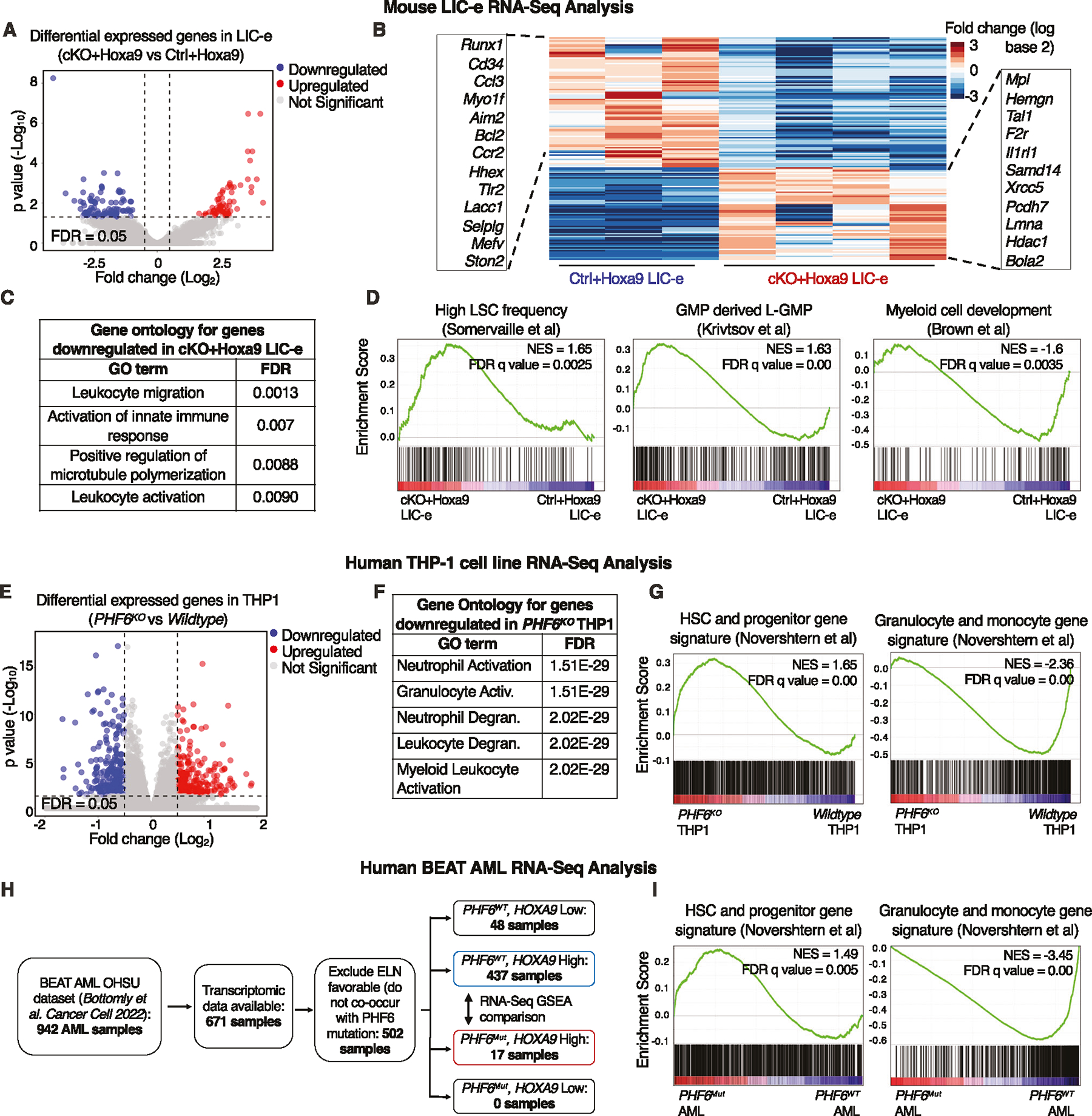
*Phf6* loss promotes a stemness gene network. **A** Volcano plot showing differentially expressed genes in LIC-e cells from *cKO*+*Hoxa9* compared to *Ctrl*+*Hoxa9* bone marrow at 8 weeks after transplantation. (n = 3–4 biological replicates). **B** Heatmap of differential expression between *Ctrl*+*Hoxa9* and *cKO*+*Hoxa9* LIC-e cells. Insets show selected downregulated (left) and upregulated (right) genes in *cKO*+*Hoxa9* LIC-e compared with *Ctrl*+*Hoxa9* LIC-e. **C** Top Gene Ontology terms enriched in genes downregulated in *cKO*+*Hoxa9* LIC-e compared with *Ctrl*+*Hoxa9* LIC-e. **D** Gene set enrichment analysis (GSEA) plots of the *cKO*+*Hoxa9* LIC-e transcriptome compared to *Ctrl*+*Hoxa9*. Plots show positive enrichment of gene sets related to high LSC frequency (left) and leukemic GMPs (middle), and negative enrichment of a gene set related to myeloid development (right). Normalized Enrichment scores (NES) and FDR q values are shown. **E** Volcano plot showing differentially expressed genes in *PHF6*^*KO*^ THP-1 cells compared to wildtype THP1 cells. (n = 4–5 clonal lines). **F** Top Gene Ontology terms enriched in genes downregulated in *PHF6*^*KO*^ THP-1 cells compared to wildtype THP1 cells. **G** Gene set enrichment analysis (GSEA) plots of the *PHF6*^*KO*^ THP-1 transcriptome compared to wildtype clones. Plots show positive enrichment of gene sets related to high HSC and Progenitors (left), and negative enrichment of a gene set related to granulocytes and monocytes (right). **H** Flow chart depicting choice of BEAT AML dataset samples picked for RNA-Seq analyses, with exclusion of cases classified as ELN favorable and those with low *HOXA9* mRNA levels (which show rare/no *PHF6* mutations). **I** Gene set enrichment analysis (GSEA) plots of the transcriptome of *PHF6* mutated AML patients with high *HOXA9* expression compared to *PHF6* wildtype AML patients with high *HOXA9* expression. Plots show positive enrichment of gene sets related to high HSC and Progenitors (left), and negative enrichment of a gene set related to granulocytes and monocytes (right).

We determined the transcriptional consequences of *Phf6* loss on *Hoxa9*-transformed marrow by performing RNA-Seq on LIC-e and committed leukemic cells from marrow of transplanted recipients at 8 weeks. Committed leukemic cells showed no change in gene expression with *Phf6* loss (Fig. S6A, B, Table S2), while the LIC-e population showed 91 downregulated and 65 upregulated genes in *cKO*+*Hoxa9* compared to *Ctrl*+*Hoxa9* (Fig. 4A, B, Table S2). Genes downregulated in *cKO*+*Hoxa9* LIC-e cells showed Gene Ontology (GO) enrichment for myeloid differentiation terms (Fig. 4C). Gene set enrichment analysis (GSEA) [31] showed that the *cKO*+*Hoxa9* LIC-e transcriptome showed positive enrichment for genesets related to high LSC potential [32] and leukemic GMPs (L-GMPs) [33] and negative enrichment for genesets related to myeloid differentiation [34], and mature neutrophils and monocytes [35] (Fig. 4D, S6C).

We next sought to determine whether *PHF6* loss produces similar transcriptional changes in human AML. We used CRISPR to generate *PHF6* knockout (*PHF6*^*KO*^) clones of the THP-1 human AML cell line (Fig. S6D). RNA-Seq showed that genes downregulated in *PHF6*^*KO*^ compared to wildtype clones were also enriched for myeloid differentiation GO terms (Fig. 4E, F, Table S3). *PHF6*^*KO*^ clones also showed positive GSEA enrichment of HSC and progenitor signatures, and negative enrichment of granulocyte and monocyte signatures [36] (Fig. 4G). We further analyzed publicly available RNA-Seq data from the BEAT AML dataset [26]. A majority of samples had high *HOXA9* mRNA levels, and *PHF6* mutated cases exclusively fell within the *HOXA9* high (*HOXA9*^*High*^) group (Fig. 4H). On comparing the transcriptomes of *PHF6* mutant (*PHF6*^*Mut*^) to *PHF6* wildtype (*PHF6*^*WT*^) AMLs within the *HOXA9*^*High*^ group, we found similar positive GSEA enrichment of HSC and progenitor signatures, and negative enrichment of granulocyte and monocyte signatures [36] (Fig. 4I). Thus, *Phf6* loss or mutation in both mouse and human AML skews their transcriptomes to a more stem-like and less differentiated state.

### *Phf6* loss prevents exhaustion of LIC-e cells by maintaining their self-renewal potential

**Fig. 5:**
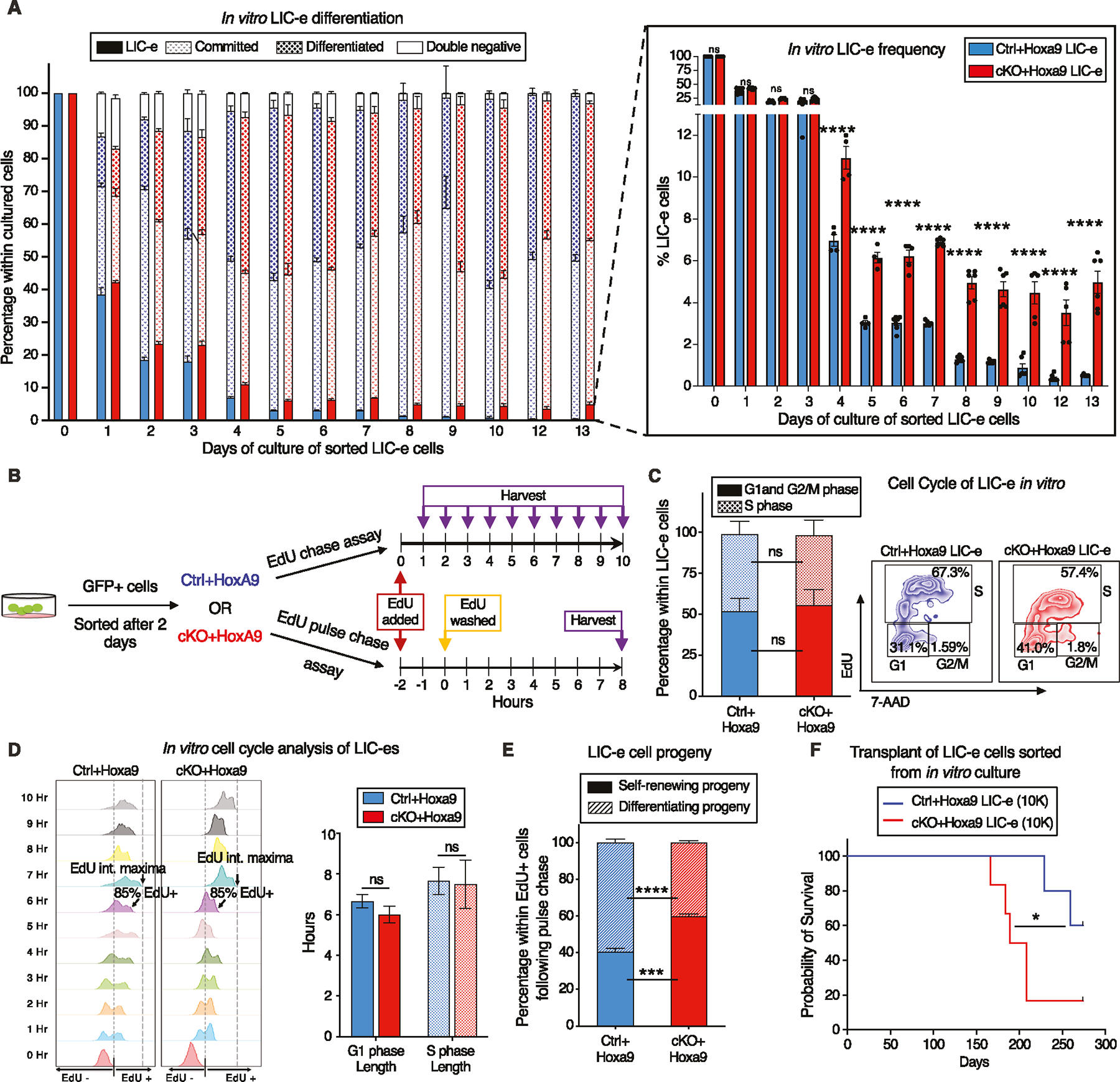
*Phf6* loss prevents exhaustion of LIC-e cells by maintaining their self-renewal potential. **A** Bar graph showing frequencies of subpopulations resulting from in vitro culture of LIC-e cells sorted 4 days after *Hoxa9* transduction of *Ctrl* and *cKO* bone marrow. Inset bar graph depicts only LIC-e frequencies in the same culture. (n = 13 biological replicates). **B** Experimental design for study of in vitro cell cycle analysis (top) and self-renewal (bottom) of LIC-e cells using EdU chase and pulse-chase assay respectively. **C** *Left*, Bar graph showing frequencies of G0/G1, S, and G2/M phases in *Ctrl*+*Hoxa9* and *cKO*+*Hoxa9* LIC-e cells in culture 2 h after addition of EdU. (n = 4–5 biological replicates) *Right*, Representative flow cytometry plots of same, with EdU marking cells in S phase and 7-AAD staining DNA. **D** *Left*, Representative flow cytometry plots showing kinetics of uptake of EdU by *Ctrl*+*Hoxa9* and *cKO*+*Hoxa9* LIC-e cells over 10 h of culture, performed for calculation of cell cycle length. Time to whole population (>85%) EdU uptake represents G1 phase length, and time to EdU intensity maxima represents S phase length. *Right*, Bar graph represents the length of cell cycle phases of LIC-e cells in culture. (n = 4–5 biological replicates) **E** Stacked bar graph showing the percentage of self-renewing and differentiating progeny produced by *Ctrl*+*Hoxa9* and *cKO*+*Hoxa9* LIC-e cells in culture. (n = 4–5 biological replicates). **F** Kaplan-Meier curve of primary transplant recipients receiving 10 K sorted *Ctrl*+*Hoxa9* and *cKO*+*Hoxa9* LIC-e cells. (n = 5–6 biological replicates) All bar graphs show mean ± SEM and statistical significance was calculated using the Student t-test. For all survival curves, statistical significance was calculated using the Log-rank (Mantel-Cox) test. *p < 0.05, **p < 0.01, ***p < 0.001; ****p < 0.0001, *ns* = *non significant*.

To determine the kinetics of the effects of *Phf6* loss on the behavior of LIC-e cells, we cultured *Hoxa9*-transduced mouse bone marrow in cytokine-supplemented media. The growth rate of bulk culture was similar for *Ctrl*+*Hoxa9* and *cKO*+*Hoxa9* marrow (Fig. S7A). When sorted LIC-e cells were cultured, most cells lost LIC-e identity within days (Fig. 5A). However, though both groups produced similar fractions of committed (c-Kit+ Ly6C+) and differentiated cells (c-Kit− Ly6C+), the *Ctrl*+*Hoxa9* culture almost completely depleted its LIC-e population (<1%), while the *cKO*+*Hoxa9* culture maintained this population, plateauing at 5–6% of the total culture after 5 days (Fig. 5A). Thus, *Phf6* loss prevents exhaustion of the LIC-e population without impairing the rate of proliferation or differentiation of the bulk culture, recapitulating the in vivo LIC-e accumulation phenotype shown earlier (Fig. 3E–G).

To reconfirm that LIC-e accumulation wasn’t due to subtle cycling differences, we performed a 10-hour cell cycle analysis in culture by adding EdU to sorted *Ctrl*+*Hoxa9* and *cKO*+*Hoxa9* LIC-e cells and harvesting them at serial time points (Fig. 5B). We observed that the distribution of *Ctrl*+*Hoxa9* or *cKO*+*Hoxa9* LIC-e cells in G1 and S phases showed no difference at the start of culture, and showed no difference in the kinetics or magnitude of EdU incorporation (Fig. 5C). Based on previously published rationale [37], we defined the time required for 85% of each sample to become EdU+ as the “G1 phase length”, and the time required for each sample to reach a maximal EdU fluorescent intensity as the ‘S phase length’. We did not observe any difference in G1 and S phase lengths between *Ctrl*+*Hoxa9* and *cKO*+*Hoxa9* LIC-e cells (Fig. 5D). We therefore hypothesized that *cKO*+*Hoxa9* LIC-e cells, while capable of producing differentiated progeny, may have a greater tendency to produce progeny with persistent LIC-e identity. To test this hypothesis, we performed an EdU pulse-chase experiment by incubating sorted LIC-e cells with EdU for 2 hours (pulse), followed by washing off the EdU and further culturing cells for 8 more hours (chase, for the length of S phase) (Fig. 5B) to allow all cells that were in S phase during the initial EdU pulse to complete mitosis, so that all EdU+ cells at the end of the 8 hour EdU-free chase would be daughter cells/progeny of the original EdU-uptaking cells. We determined the percentage of self-renewing progeny by calculating the percentage of total progeny (total EdU+ cells) that had LIC-e markers (Ly6C−EdU+), while the rest (Ly6C+ EdU+) were differentiating progeny. We observed that while 40.6% of the progeny of *Ctrl*+*Hoxa9* LIC-e cells were also LIC-e cells, this fraction was increased to 60.0% in *cKO*+*Hoxa9* (Fig. 5E). To confirm that in vitro LIC-e cells are functionally equivalent to their in vivo counterparts, we performed primary transplantation with 10 K LIC-e cells sorted after 2 days of culture. Recipients of *cKO*+*Hoxa9* LIC-e cells succumbed faster than *Ctrl*+*Hoxa9* LIC-e (Fig. 5F). Collectively, *Phf6* loss prevents the exhaustion of LIC-e cells by increasing the fraction of their progeny that retain persistent LIC-e identity.

### Effects of Phf6 loss on chromatin accessibility in LIC-e cells

**Fig. 6:**
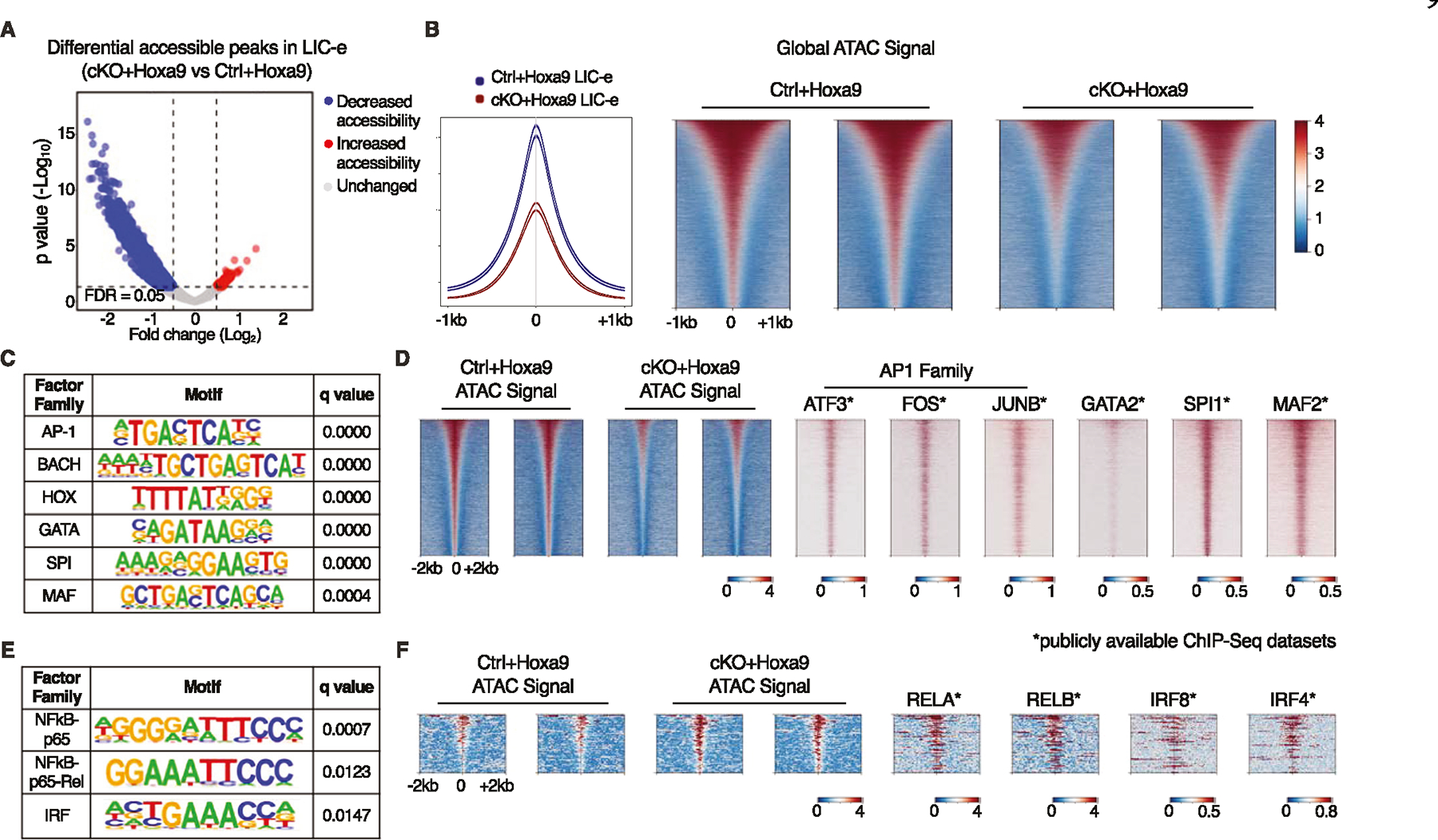
Effects of Phf6 loss on chromatin accessibility in LIC-e cells. **A** Volcano plot showing differentially accessible regions in LIC-e cells from *cKO*+*Hoxa9* compared *Ctrl*+*Hoxa9*. (n = 3 biological replicates). **B** Representative signal profile (left) and metagene plots (right) showing genome-wide intensity of ATAC signal in *Ctrl*+*Hoxa9* and *cKO*+*Hoxa9* LIC-e cells. **C** HOMER analysis for regions of decreased chromatin accessibility in *cKO*+*Hoxa9* LIC-e cells, showing enrichment of AP-1, HOX, GATA, SPI1, and MAF motifs. **D** Representative metagene plots at regions of decreased chromatin accessibility in *cKO*+*Hoxa9* LIC-e, show ChIP-Seq signal for select proteins whose motifs are seen to be enriched through HOMER in (**C**). **E** HOMER analysis for regions of increased chromatin accessibility in *cKO*+*Hoxa9* LIC-e cells showing enrichment of NF-kB and IRF motifs. **F** Representative metagene plots at regions of increased chromatin accessibility in *cKO*+*Hoxa9* LIC-e, show ChIP-Seq signal for select proteins whose motifs are seen to be enriched through HOMER in (**E**). Publicly available ChIP-Seq datasets in leukemia or myeloid cells were used in metagene heatmaps (Table S4). All plots were centered around ATAC-Seq peaks. SeqPlots was used to draw all metagene plots.

To profile the effects of *Phf6* loss on the accessibility landscape of LIC-e cells, we performed ATAC-Seq on sorted LIC-e cells from freshly transduced *cKO*+*Hoxa9* and *Ctrl*+*Hoxa9* marrow. We observed that *cKO*+*Hoxa9* LIC-e cells showed a global reduction in chromatin accessibility compared to *Ctrl*+*Hoxa9*, with only a few regions showing increased accessibility (Fig. 6A, B). Regions that lost accessibility in *cKO*+*Hoxa9* LIC-e cells showed enrichment for AP-1, HOX, SPI, and GATA family motifs, among others (Fig. 6C). Public ChIP-Seq tracks from leukemic or myeloid cells showed co-occupancy of these factors at ATAC peaks with reduced accessibility (Fig. 6D, Table S4). Promoters of multiple genes like *Runx1, Selplg*, and *Aim2*, which are downregulated in *cKO*+*Hoxa9* LIC-e cells (Fig. 4B), showed occupancy by these factors and showed reduced chromatin accessibility in *cKO*+*Hoxa9* LIC-e cells (Fig. S7B). Conversely, the small number of regions that gained accessibility in *cKO*+*Hoxa9* LIC-e cells showed enrichment for NF-kB and IRF family motifs (Fig. 6E). Public ChIP-Seq tracks showed co-occupancy of NF-kB (RELA, RELB) and IRF8, IRF4 at regions of increased accessibility (Fig. 6F, Table S4). Overall, *Phf6* loss, likely via a combination of direct and indirect effects, led to altered accessibility at sites bound by key hematopoietic transcription factors.

## DISCUSSION

In concordance with the genomic observation that majority of *PHF6* somatic mutations are presumed loss-of-function frameshift and nonsense mutations, multiple mouse studies have reported increased HSC self-renewal with *Phf6* knockout [17–19, 27], and have reported enhanced T-ALL progression when *Phf6* knockout is combined with activating mutations in *Notch1* or *Jak3*, or overexpression of *Tlx3* [17, 18, 20]. In contrast, a recent publication (Hou et al. [23]) reported that AML induced by *BCR-ABL, AML1-ETO*, and *MLL-AF9* fusions is impaired by *Phf6* loss, reaching the counterintuitive conclusion that *Phf6* is required for myeloid leukemogenesis. Our paper addresses this controversy, and we demonstrate that *Phf6* loss accelerates AML in a broadly relevant model and that it does so by increasing the frequency and persistence of leukemic stem cells, a finding that is harmonious with the known role of *Phf6* as a repressor of HSC self-renewal.

We first interrogated the BEAT AML dataset, which showed that *PHF6* mutations are associated with worsened survival in human AML. Then, by using the mouse *Hoxa9* transduction AML model, we demonstrated that *Phf6* loss led to increased colony replating, increased disease burden in vivo, progressively worsened survival on serial transplantation, and increased LIC frequency. We determined that a simplified gating scheme could identify an LIC-enriched population (LIC-e), which was the only population capable of robust colony plating and engraftment. We found that *Phf6* loss led to an expansion of LIC-e cells and that this expansion could be recapitulated in vitro. Contrary to reports that *Phf6* loss alters cell cycle or apoptosis, we found evidence of neither, and instead found that *Phf6* loss leads LIC-e cells to produce more progeny with persistent LIC-e identity, thus indicating that *Phf6* specifically controls the balance between LIC self-renewal and differentiation. Our RNA-Seq analyses of mouse LIC-e cells, as well as of human THP-1 AML cells and primary patient samples from BEAT AML, show that *PHF6* loss consistently skews the transcriptome to a more stem-like state.

There could be multiple reasons for why our results stand in contrast to those of Hou et al. First, we specifically picked *Hoxa9* transduction as a driver that broadly recapitulates AML biology, while Hou et al used fusions that are not known to co-occur with *PHF6* in patients. It is therefore unclear if their models reflected the in vivo context in which *PHF6* mutations gain a clonal advantage in humans. Notably, we found that *Phf6* knockout could not further accelerate the already rapid proliferation and near-inexhaustible replating capacity of *MLL-AF9*-overexpressing marrow, further supporting the use of the slow-kinetic *Hoxa9* model. Second, though both our groups used *Vav-Cre* to knock out *Phf6*, Hou et al. used *Flox*-only mice as negative controls, while we used *Vav-Cre*-only mice. *Cre* toxicity has been reported with other *Cre* models [38, 39], and may be a contributory factor to these discordant phenotypes; our approach eliminates *Cre* toxicity as a potential confounder. Third, it is possible that *Phf6* loss produces divergent effects in certain AML contexts compared to others, and further work in human genomics and mouse modeling will be required to define the specific co-mutational contexts in which *Phf6* loss affects AML stemness and growth.

We observed a striking reduction in global chromatin accessibility in LIC-e cells on *Phf6* loss, with a third of all peaks showing significantly reduced ATAC signal. This translated into reduced accessibility at sites with AP-1 family, GATA2, and SPI1 occupancy, and increased accessibility at a small number of sites with NF-kB and IRF family occupancy. This observation does not allow us to draw any immediate conclusion about the direct molecular action of PHF6 protein on chromatin, and we believe that our results reflect a composite of indirect and cumulative effects in an LIC-e population that is not epigenetically homogenous. Future work using bulk and single-cell ATAC-Seq studies will be required to map out the precise chromatin effects of *Phf6* loss in the *Hoxa9* model, as well as in other models such as co-mutation with *RUNX1* or *ASXL1*.

In summary, our work defines an LIC-enriched population in *Hoxa9*-driven AML, and the hierarchy through which it differentiates and expands to produce the bulk of the AML population. We show that *Phf6* loss increases the number of these LIC-e cells, not through increased cycling, but by producing progeny with persistent LIC-e identity. Taken together with the relatively pure phenotype of increased HSC self-renewal observed when *Phf6* is knocked out in homeostatic marrow, this presents a useful system to demonstrate how the normal process of self-renewal is co-opted in AML to drive the self-renewal of leukemic stem cells.

## MATERIALS AND METHODS

Cryopreserved sperm from *Phf6*^*fl/Y*^ mice (serial# 4621–2 / G4621) was purchased from the Mouse Clinical Institute at GIE-CERBM (GIE-Centre Européen de Recherche en Biologie et Médecine, France), and pups were generated by the Children’s Hospital of Philadelphia Transgenic Core by IVF using C57BL/6J oocytes. *Vav-Cre* mice were originally generated by Thomas Graf [40] and were provided as a generous gift by Warren Pear (Department of Pathology, University of Pennsylvania Perelman School of Medicine). Both alleles were backcrossed with pure C57BL/6J for over 10 generations. All animals were maintained and experiments were carried out according to the University of Pennsylvania’s Animal Resources Center and IACUC protocols.

Please refer to supplemental data for additional methods.

## Supplementary Material

Supplemental Figures

Supplemental Methods

Table S1

Table S2

Table S3

Table S4

## Data Availability

All generated datasets have been deposited to GEO: GSE270756.

## References

[1] Zhang C The X-linked intellectual disability protein PHF6 associates with the PAF1 complex and regulates neuronal migration in the mammalian brain. Neuron. 2013;78:986–93. 23791194 10.1016/j.neuron.2013.04.021 PMC3694281

[2] Wang J, Leung JW-C, Gong Z, Feng L, Shi X, Chen J. PHF6 regulates cell cycle progression by suppressing ribosomal RNA synthesis. J Biol Chem. 2013;288:3174–83. 23229552 10.1074/jbc.M112.414839 PMC3561539

[3] Warmerdam DO, Alonso-de Vega I, Wiegant WW, van den Broek B, Rother MB, Wolthuis RM, PHF6 promotes non-homologous end joining and G2 checkpoint recovery. EMBO Rep. 2020;21:e48460. 31782600 10.15252/embr.201948460 PMC6944915

[4] Wendorff AA, Aidan Quinn S, Alvarez S, Brown JA, Biswas M, Gunning T, Epigenetic reversal of hematopoietic stem cell aging in Phf6-knockout mice. Nat Aging. 2022;2:1008–23. 37118089 10.1038/s43587-022-00304-x PMC12077290

[5] Soto-Feliciano YM, Bartlebaugh JME, Liu Y, Sánchez-Rivera FJ, Bhutkar A, Weintraub AS, PHF6 regulates phenotypic plasticity through chromatin organization within lineage-specific genes. Genes Dev. 2017;31:973–89. 28607179 10.1101/gad.295857.117 PMC5495126

[6] Todd MAM, Picketts DJ. PHF6 interacts with the nucleosome remodeling and deacetylation (NuRD) complex. J Proteome Res. 2012;11:4326–37. 22720776 10.1021/pr3004369

[7] Alvarez S, da Silva Almeida AC, Albero R, Biswas M, Barreto-Galvez A, Gunning TS, Functional mapping of PHF6 complexes in chromatin remodeling, replication dynamics, and DNA repair. Blood. 2022;139:3418–29. 35338774 10.1182/blood.2021014103 PMC9185155

[8] Oh S, Boo K, Kim J, Baek SA, Jeon Y, You J, The chromatin-binding protein PHF6 functions as an E3 ubiquitin ligase of H2BK120 via H2BK12Ac recognition for activation of trophectodermal genes. Nucleic Acids Res. 2020;48:9037–52. 32735658 10.1093/nar/gkaa626 PMC7498345

[9] Van Vlierberghe P, Palomero T, Khiabanian H, Van der Meulen J, Castillo M, Van Roy N, PHF6 mutations in T-cell acute lymphoblastic leukemia. Nat Genet. 2010;42:338–42. 20228800 10.1038/ng.542 PMC2847364

[10] Van Vlierberghe P, Patel J, Abdel-Wahab O, Lobry C, Hedvat CV, Balbin M, PHF6 mutations in adult acute myeloid leukemia. Leukemia. 2011;25:130–4. 21030981 10.1038/leu.2010.247 PMC3878659

[11] Patel JP, Gönen M, Figueroa ME, Fernandez H, Sun Z, Racevskis J, Prognostic relevance of integrated genetic profiling in acute myeloid leukemia. N Engl J Med. 2012;366:1079–89. 22417203 10.1056/NEJMoa1112304 PMC3545649

[12] Xiao W, Bharadwaj M, Levine M, Farnhoud N, Pastore F, Getta BM, PHF6 and DNMT3A mutations are enriched in distinct subgroups of mixed phenotype acute leukemia with T-lineage differentiation. Blood Adv. 2018;2:3526–39.. 30530780 10.1182/bloodadvances.2018023531 PMC6290101

[13] Papaemmanuil E, Gerstung M, Bullinger L, Gaidzik VI, Paschka P, Roberts ND, Genomic classification and prognosis in acute myeloid leukemia. N Engl J Med. 2016;374:2209–21. 27276561 10.1056/NEJMoa1516192 PMC4979995

[14] Bataller A, Chien KS, Sasaki K, Montalban-Bravo G, Kanagal-Shamanna R, Urrutia S, PHF6 mutations in myelodysplastic syndromes, chronic myelomonocytic leukemia and acute myeloid leukemia. Leuk Res. 2023;127:107044. 36801700 10.1016/j.leukres.2023.107044

[15] Huang K, Wang L, Zheng Y, Yue C, Xu X, Chen H, PHF6 mutation is associated with poor outcome in acute myeloid leukaemia. Cancer Med. 2023;12:2795–804. 36176187 10.1002/cam4.5173 PMC9939093

[16] Cerami E, Gao J, Dogrusoz U, Gross BE, Sumer SO, Aksoy BA, The cBio cancer genomics portal: an open platform for exploring multidimensional cancer genomics data. Cancer Discov. 2012;2:401–4. 22588877 10.1158/2159-8290.CD-12-0095 PMC3956037

[17] McRae HM, Garnham AL, Hu Y, Witkowski MT, Corbett MA, Dixon MP, PHF6 regulates hematopoietic stem and progenitor cells and its loss synergizes with expression of TLX3 to cause leukemia. Blood. 2019;133:1729–41. 30755422 10.1182/blood-2018-07-860726 PMC6695515

[18] Wendorff AA, Quinn SA, Rashkovan M, Madubata CJ, Ambesi-Impiombato A, Litzow MR, Phf6loss enhances HSC self-renewal driving tumor initiation and leukemia stem cell activity in T-ALL. Cancer Discov. 2019;9:436–51. 30567843 10.1158/2159-8290.CD-18-1005 PMC6425751

[19] Hsu Y-C, Chen T-C, Lin C-C, Yuan C-T, Hsu C-L, Hou H-A, Phf6-null hematopoietic stem cells have enhanced self-renewal capacity and oncogenic potentials. Blood Adv. 2019;3:2355–67. 31395598 10.1182/bloodadvances.2019000391 PMC6693005

[20] Yuan S, Wang X, Hou S, Guo T, Lan Y, Yang S, PHF6 and JAK3 mutations cooperate to drive T-cell acute lymphoblastic leukemia progression. Leukemia. 2022;36:370–82. 34465864 10.1038/s41375-021-01392-1 PMC8807395

[21] Chen T-C, Yao C-Y, Chen Y-R, Yuan C-T, Lin C-C, Hsu Y-C, Oncogenesis induced by combined Phf6 and Idh2 mutations through increased oncometabolites and impaired DNA repair. Oncogene. 2022;41:1576–88. 35091680 10.1038/s41388-022-02193-1

[22] Meacham CE, Lawton LN, Soto-Feliciano YM, Pritchard JR, Joughin BA, Ehrenberger T, A genome-scale in vivo loss-of-function screen identifies Phf6 as a lineage-specific regulator of leukemia cell growth. Genes Dev. 2015;29:483–8. 25737277 10.1101/gad.254151.114 PMC4358400

[23] Hou S, Wang X, Guo T, Lan Y, Yuan S, Yang S, PHF6 maintains acute myeloid leukemia via regulating NF-κB signaling pathway. Leukemia. 2023;37:1626–37. 37393343 10.1038/s41375-023-01953-6 PMC10400421

[24] Kroon E, Krosl J, Thorsteinsdottir U, Baban S, Buchberg AM, Sauvageau G. Hoxa9 transforms primary bone marrow cells through specific collaboration with Meis1a but not Pbx1b. EMBO J. 1998;17:3714–25. 9649441 10.1093/emboj/17.13.3714 PMC1170707

[25] Spencer DH, Young MA, Lamprecht TL, Helton NM, Fulton R, O’Laughlin M, Epigenomic analysis of the HOX gene loci reveals mechanisms that may control canonical expression patterns in AML and normal hematopoietic cells. Leukemia. 2015;29:1279–89. 25600023 10.1038/leu.2015.6 PMC4456213

[26] Bottomly D, Long N, Schultz AR, Kurtz SE, Tognon CE, Johnson K, Integrative analysis of drug response and clinical outcome in acute myeloid leukemia. Cancer Cell. 2022;40:850–.e9. 35868306 10.1016/j.ccell.2022.07.002 PMC9378589

[27] Miyagi S, Sroczynska P, Kato Y, Nakajima-Takagi Y, Oshima M, Rizq O, The chromatin-binding protein Phf6 restricts the self-renewal of hematopoietic stem cells. Blood. 2019;133:2495–506. 30917958 10.1182/blood.2019000468

[28] Spring J, Khan AA, Lara S, O’Grady K, Wilks J, Gurbuxani S, Gut commensal bacteria enhance pathogenesis of a tumorigenic murine retrovirus. Cell Rep. 2022;40:111341. 36103821 10.1016/j.celrep.2022.111341 PMC10226680

[29] Ikeda H, Kanakura Y, Tamaki T, Kuriu A, Kitayama H, Ishikawa J, Expression and functional role of the proto-oncogene c-kit in acute myeloblastic leukemia cells. Blood. 1991;78:2962–8. 1720040

[30] Somervaille TCP, Cleary ML. Identification and characterization of leukemia stem cells in murine MLL-AF9 acute myeloid leukemia. Cancer Cell. 2006;10:257–68. 17045204 10.1016/j.ccr.2006.08.020

[31] Subramanian A, Kuehn H, Gould J, Tamayo P, Mesirov JP. GSEA-P: a desktop application for gene set enrichment analysis. Bioinformatics. 2007;23:3251–3. 17644558 10.1093/bioinformatics/btm369

[32] Somervaille TCP, Matheny CJ, Spencer GJ, Iwasaki M, Rinn JL, Witten DM, Hierarchical maintenance of MLL myeloid leukemia stem cells employs a transcriptional program shared with embryonic rather than adult stem cells. Cell Stem Cell. 2009;4:129–40. 19200802 10.1016/j.stem.2008.11.015 PMC2670853

[33] Krivtsov AV, Twomey D, Feng Z, Stubbs MC, Wang Y, Faber J, Transformation from committed progenitor to leukaemia stem cell initiated by MLL–AF9. Nature. 2006;442:818–22. 16862118 10.1038/nature04980

[34] Brown AL, Wilkinson CR, Waterman SR, Kok CH, Salerno DG, Diakiw SM, Genetic regulators of myelopoiesis and leukemic signaling identified by gene profiling and linear modeling. J Leukoc Biol. 2006;80:433–47. 16769770 10.1189/jlb.0206112

[35] Franzén O, Gan L-M, Björkegren JLM. PanglaoDB: a web server for exploration of mouse and human single-cell RNA sequencing data. Database. 2019;2019:baz046. 30951143 10.1093/database/baz046 PMC6450036

[36] Novershtern N, Subramanian A, Lawton LN, Mak RH, Haining WN, McConkey ME, Densely interconnected transcriptional circuits control cell states in human hematopoiesis. Cell. 2011;144:296–309. 21241896 10.1016/j.cell.2011.01.004 PMC3049864

[37] Pereira PD, Serra-Caetano A, Cabrita M, Bekman E, Braga J, Rino J, Quantification of cell cycle kinetics by EdU (5-ethynyl-2’-deoxyuridine)-coupled-fluorescence-intensity analysis. Oncotarget. 2017;8:40514–32. 28465489 10.18632/oncotarget.17121 PMC5522303

[38] Higashi AY, Ikawa T, Muramatsu M, Economides AN, Niwa A, Okuda T, Direct hematological toxicity and illegitimate chromosomal recombination caused by the systemic activation of CreERT2. J Immunol. 2009;182:5633–40. 19380810 10.4049/jimmunol.0802413

[39] Naiche LA, Papaioannou VE. Cre activity causes widespread apoptosis and lethal anemia during embryonic development. Genesis. 2007;45:768–75. 18064676 10.1002/dvg.20353

[40] Stadtfeld M, Graf T. Assessing the role of hematopoietic plasticity for endothelial and hepatocyte development by non-invasive lineage tracing. Development. 2005;132:203–13. 15576407 10.1242/dev.01558

[41] O’Connell KE, Mikkola AM, Stepanek AM, Vernet A, Hall CD, Sun CC, Practical murine hematopathology: a comparative review and implications for research. Comp Med. 2015;65:96–113. 25926395 PMC4408895

