# Supplementary material for: PHF6 suppresses self-renewal of leukemic stem cells in AML
Source: Leukemia. Author manuscript; Available in PMC 2024 Sep 1. (PMC11347380; doi:10.1038/s41375-024-02340-5)
Supplement: Supplemental Figures [file NIHMS2009447-supplement-Supplemental_Figures.pdf]

Jalnapurkar et al, Figure S1

A

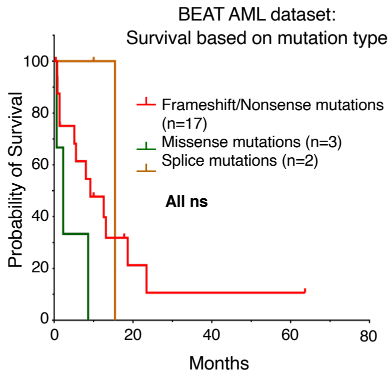

B

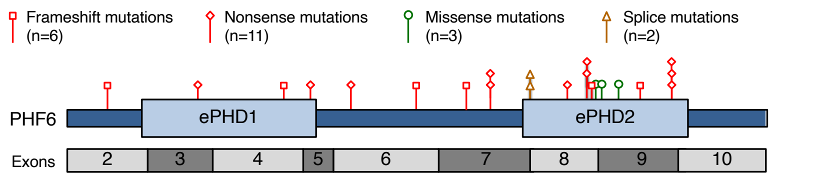

C

Mutation	Mutation Type	ELN Category	Overall Survival (Months)
H302Ifs*49	Frameshift	Intermediate	23.4
R277Efs*2	Frameshift	Adverse	0.8
H185Qfs*5	Frameshift	Adverse	5.1
C212Wfs*8	Frameshift	N/A (Relapse Sample)	5.6
Q114Hfs*30	Frameshift	N/A (Relapse Sample)	13.2
N23Kfs*3	Frameshift	N/A (Relapse Sample)	18.7
R274*	Nonsense	N/A (Relapse Sample)	9.2
Q71*	Nonsense	N/A (Relapse Sample)	12.6
R225*	Nonsense	Adverse	8.1
R225*	Nonsense	N/A (Relapse Sample)	10.0*
R319*	Nonsense	Adverse	1.4
R319*	Nonsense	Adverse	1.4
D264Gfs*3	Nonsense	Intermediate	1.5*
R319*	Nonsense	Adverse	0.9
R274*	Nonsense	Intermediate	63.6*
E150*	Nonsense	Adverse	0.4*
R129*	Nonsense	Adverse	17.8*
C283S	Missense	Adverse	2.3
G291E	Missense	Adverse	8.6
C280Y	Missense	Adverse	0.7
X244_splice	Splice	Adverse	15.5
X244_splice	Splice	N/A (Relapse Sample)	10.0*

*represents points censored for survival analysis

D

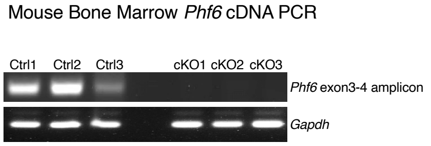

E

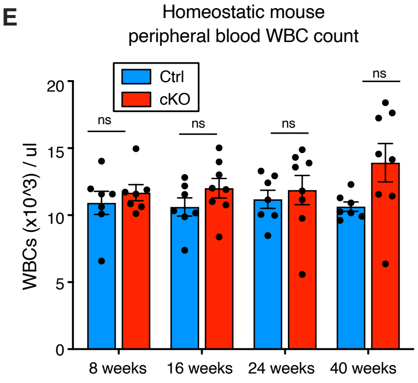

F G H I J K

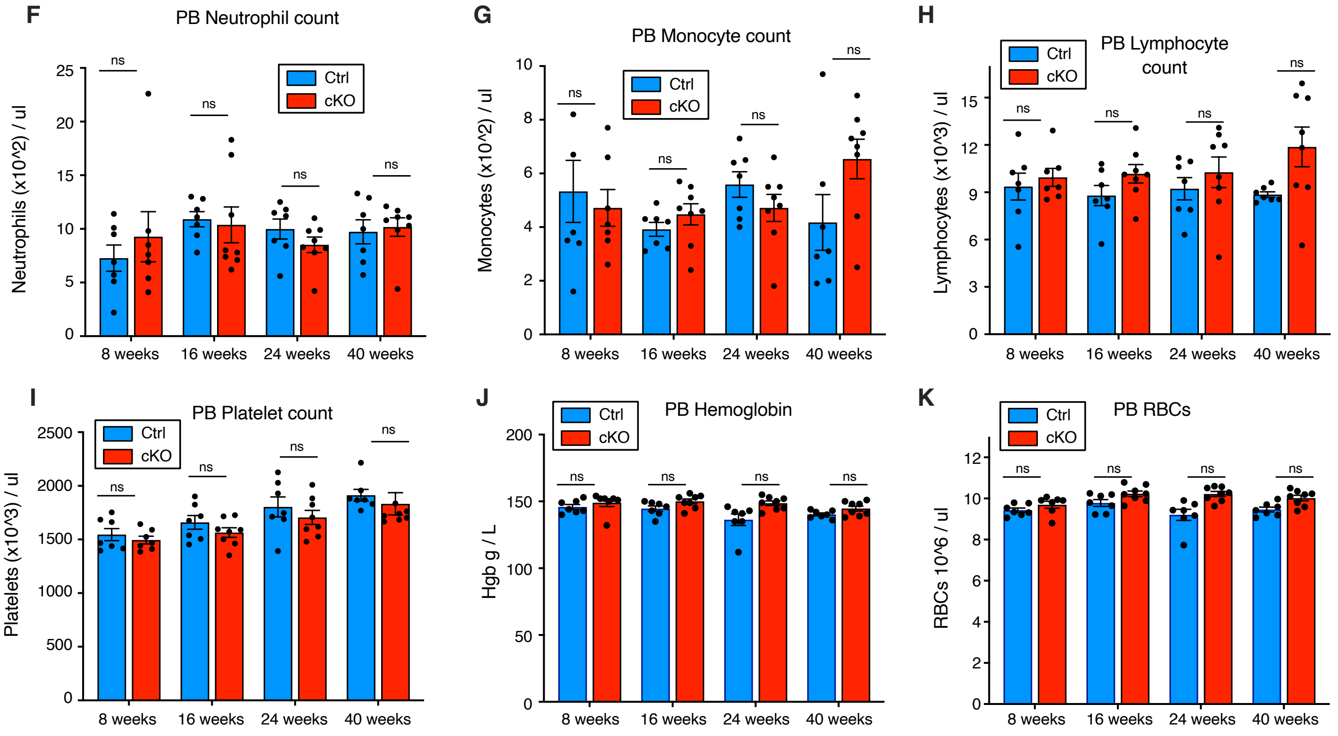

Figure S1 (related to Figure 1).

A, Kaplan-Meier survival curves for *PHF6* mutated patients according to mutation type in adverse and intermediate risk (ELN2017 classification) adult AML patients from the BEAT AML dataset. Statistical significance was calculated using the Log-rank (Mantel-Cox) test.

B, Lillipop mutation diagram representing different types of *PHF6* mutations in adverse, intermediate risk, and non-initiating (ELN2017 classification) adult AML patients from the BEAT AML dataset.

C, Tabular representation of different types of *PHF6* mutations in adverse and intermediate risk (ELN classification) adult AML patients from the BEAT AML dataset.

D, *Phf6* mRNA expression in bone marrow of *Vav-Cre*$^{Cre/+}$ *Phf6*$^{+/y}$ (*Ctrl*) and *Vav-Cre*$^{Cre/+}$ *Phf6*$^{fl/y}$ (*cKO*) mice. *Gapdh* is shown as a loading control. n = 3 biological replicates

E-K, Bar graphs depicting peripheral blood counts for WBCs, neutrophils, lymphocytes, monocytes, platelets, hemoglobin and RBCs for *Ctrl* and *cKO* mice at 8, 16, 24, and 40 weeks of age. n = 8 biological replicates

All bar graphs show mean ± SEM. n.s. = non-significant by student t-test.

Jalnapurkar et al, Figure S2

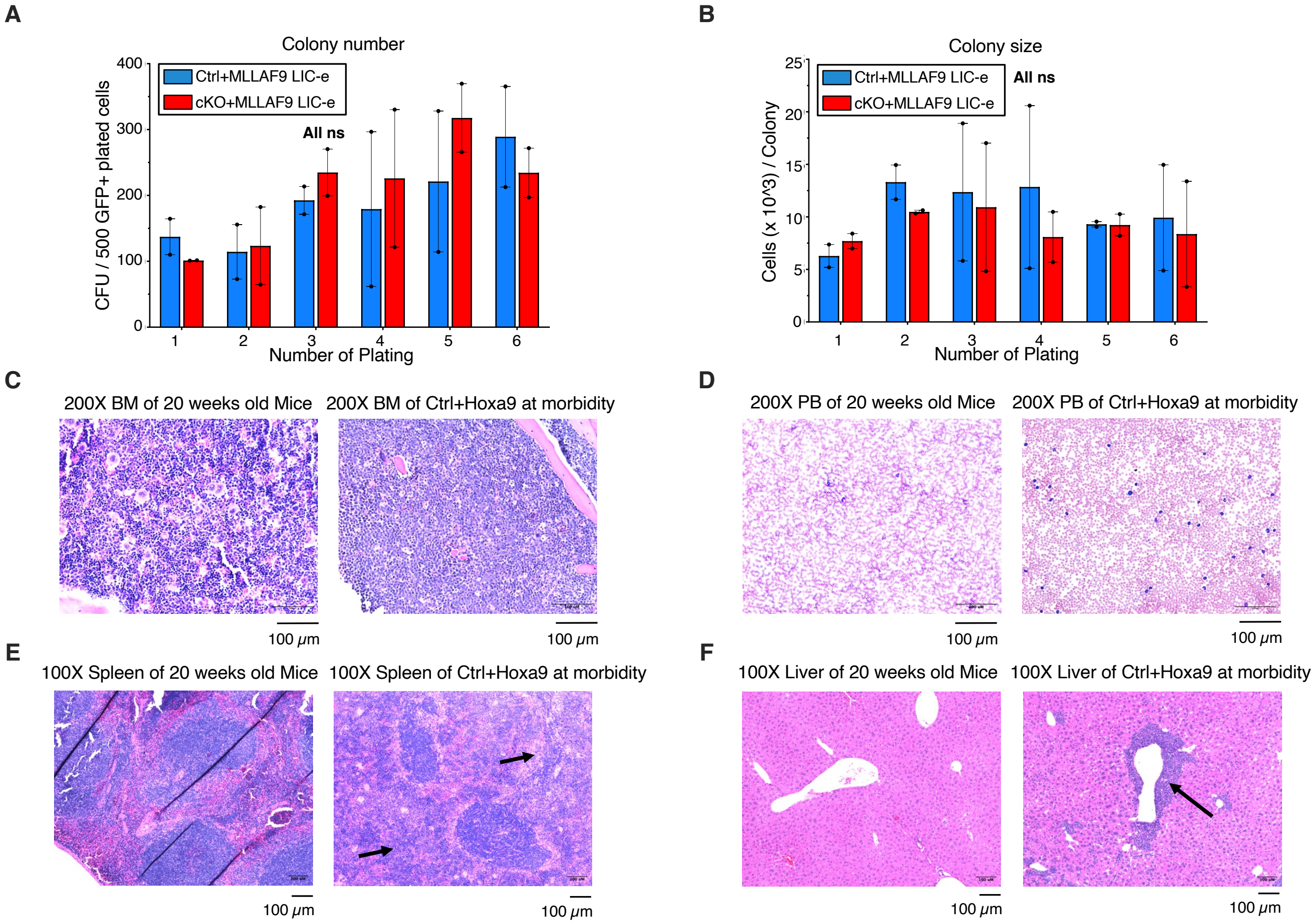

Figure S2 (related to Figure 1).

A, Bar graph showing number of colony forming units (CFUs) obtained from 6 rounds of serial methylcellulose replating of 500 cells/plate of Ctrl+MLL-AF9 and cKO+MLL-AF9 transformed mouse bone marrow. (n = 2 biological replicates)

B, Bar graph showing average number of cells per colony (colony size) obtained after 6 rounds of serial methylcellulose replating of 500 cells/plate of Ctrl+MLL-AF9 and cKO+MLL-AF9 transformed mouse bone marrow. (n = 2 biological replicates).

C, H&E staining of bone marrow collected from a representative *Ctrl+Hoxa9* mouse at morbidity, with age-matched homeostatic (non-leukemic) WT mouse shown for reference. Scale bar is 100um at 200X.

D, Wright-Giemsa stain of blood smears from a representative *Ctrl+Hoxa9* mouse at morbidity, with age-matched homeostatic WT mouse shown for reference. Scale bar is 100um at 200X.

E-F, H&E staining of (**E**) spleen and (**F**) liver collected from a representative *Ctrl+Hoxa9* mouse at morbidity, with age-matched homeostatic (non-leukemic) WT mouse shown for reference. Scale bar is 100um at 100X. Arrows indicate leukemic infiltration.

All bar graphs show mean ± SEM. n.s. = non-significant by student t-test.

Jalnapurkar et al, Figure S3

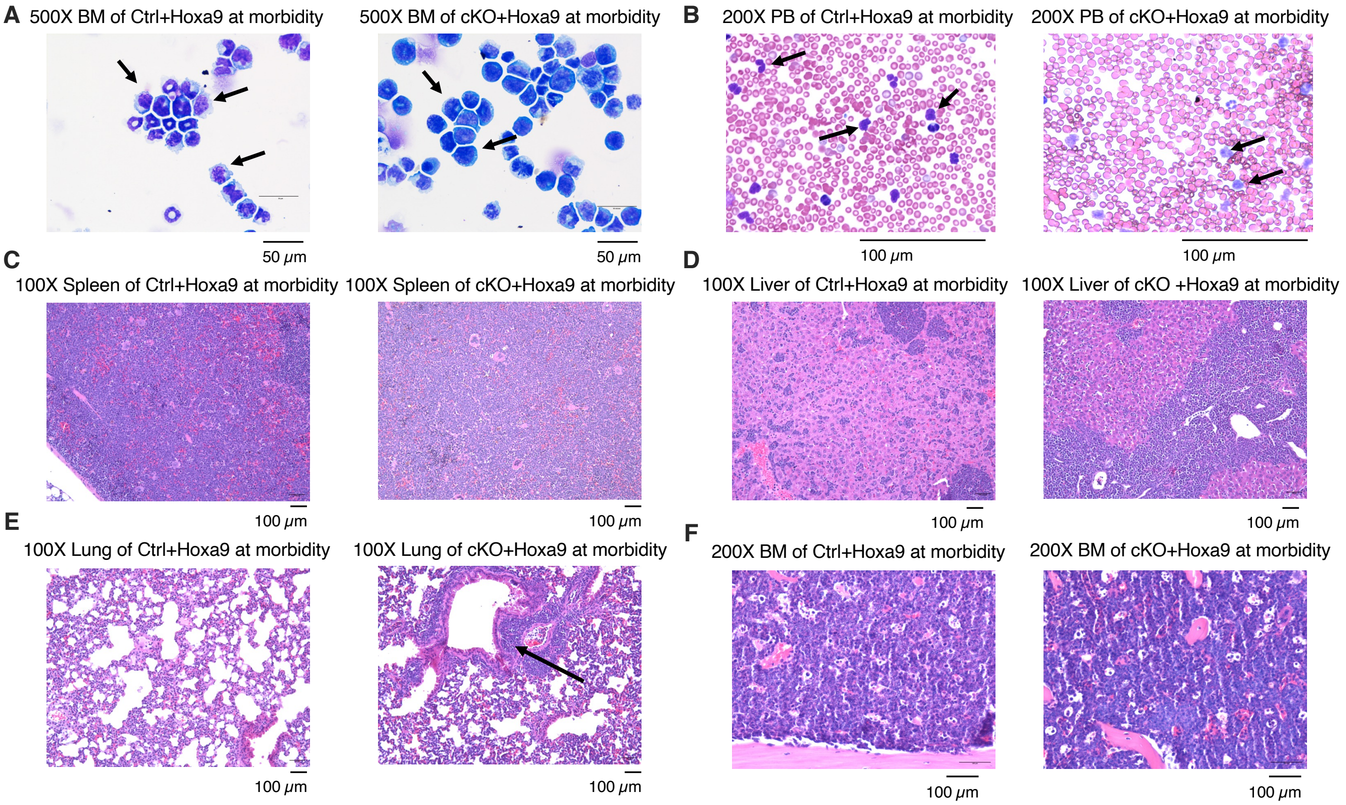

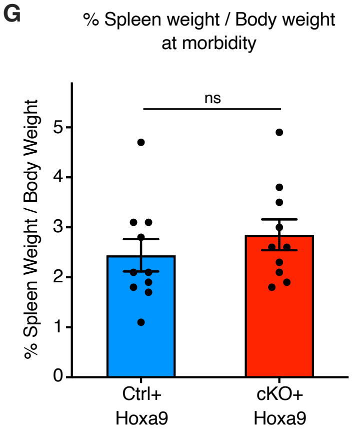

H

Transplant	Dose	Ctrl+Hoxa9	cKO+Hoxa9	p value
Primary	400K	96.5 days	112 days	0.391
	100K	142 days	114 days	0.795
	30K	268 days	225 days	0.0793
Secondary	100K	163 days	93 days	0.0511
	30K	160 days	106 days	0.0011
Tertiary	100K	117 days	57 days	<0.0001

Figure S3 (related to Figure 1). Loss of Phf6 accelerates HoxA9-driven AML on serial transplantation

A, Representative image of Wright-Giemsa stain of bone marrow cytospins of *Ctrl+Hoxa9* and *cKO+Hoxa9* mice at morbidity. Scale bar is 50um at 500X. Arrows indicate blast cells.

B, Representative image of Wright-Giemsa stain of blood smears of *Ctrl+Hoxa9* mouse and *cKO+Hoxa9* mouse at morbidity. Scale bar is 100um at 200X. Arrows indicate blast cells.

C-F, Representative image of H&E staining of (**C**) spleen, (**D**) liver, (**E**) lung, and (**F**) bone marrow collected from *Ctrl+Hoxa9* mouse and *cKO+Hoxa9* mouse at morbidity. Scale bar is 100uM at 100X. Black arrows indicate leukemic infiltration.

G, Bar graph showing percent spleen / body weight at morbidity. Statistical significance was calculated using the Student t test. n.s. = non-significant by student t-test.

H, Table with median survival and statistical significance for primary, secondary and tertiary transplantation with different dose of *Ctrl+Hoxa9* and *cKO+Hoxa9* cells.

Jalnapurkar et al, Figure S4

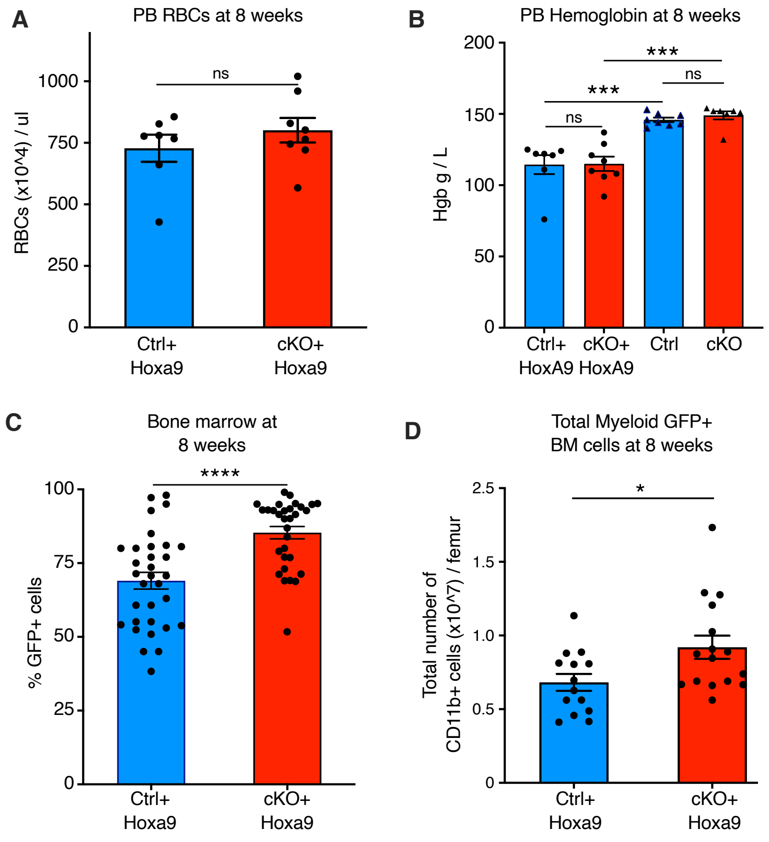

Figure S4 (related to Figure 2). ***Phf6*** **loss increases leukemic disease burden**

A-B, Bar graphs of peripheral blood counts for (**A**) RBCs and (**B**) hemoglobin level of *Ctrl+Hoxa9* and *cKO+Hoxa9* mice at 8 weeks, post primary transplantation. Hemoglobin levels of homeostatic (non-leukemic, non-transplanted) *Ctrl* and *cKO* mice are shown for reference. n = 7 biological replicates, n.s. = non-significant by student t-test.

C-D, Frequency of (**C**) GFP+ cells per femur and (**D**) total number of myeloid GFP+ cells per femur in the bone marrow of primary recipient after 8 weeks of transplantation of *Ctrl+Hoxa9* and *cKO+Hoxa9* cells.

Jalnapurkar et al, Figure S5

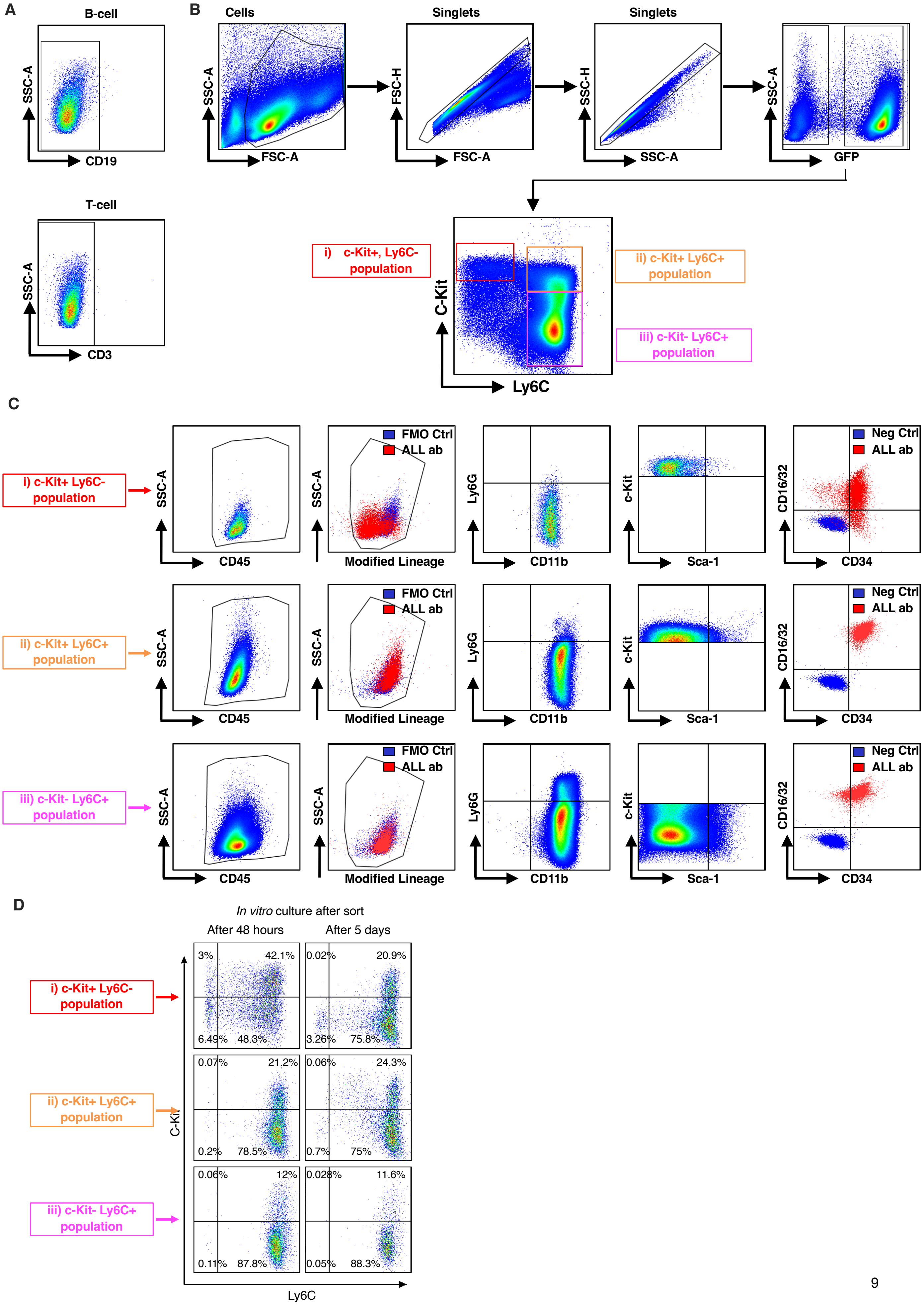

Figure S5 (related to Figure 3). Phenotypic characterization of *Hoxa9*-driven AML subpopulations

A, Representative FACS plot of bone marrow leukemic cells (GFP+) from primary recipient mice at 8 weeks after transplantation. FACS plots demonstrate absence of B cell specific (CD19) and T cell specific (CD3) antigen expression.

B-C, FACS plots demonstrating the gating strategy used to compartmentalized GFP+ cells. We have designated the three sub-populations: red (c-Kit+ Ly6C-), orange (c-Kit+ Ly6C+) and pink (c-Kit- Ly6C+) as LIC-e, Committed and Differentiated leukemic cells respectively. Each population is further characterized for the expression of CD45 (pan hematopoietic marker), modified non-myeloid lineage cocktail (CD3, CD19, CD45R, Ter119, CD49b), CD11b (myeloid marker), Ly6G (neutrophil marker), c-kit, Sca-1, CD34, and CD16/32.

LIC-e cells were: $mLin^{-}Kit^{+}Sca^{-}$ $CD11b^{dim}$ $Ly6G^{-}$ $CD34^{+}$ $CD16/32^{-/+}$ $Ly6C^{-}$

Committed cells were: $mLin^{-}Kit^{+}Sca^{-}$ $CD11b^{+}$ $Ly6G^{-/+}$ $CD34^{+}$ $CD16/32^{+}$ $Ly6C^{+}$

Differentiated cells were: $mLin^{-}Kit^{-}Sca^{-}$ $CD11b^{+}$ $Ly6G^{-/+}$ $CD34^{+}$ $CD16/32^{+}$ $Ly6C^{+}$

Note: The main flow plot in Fig S4B is also shown in main Fig 3A (shown here with detailed gating strategy).

D, Representative flow cytometry plots showing immunophenotypes of cells obtained after 48 hours and 5 days of *in vitro* culture of the three sorted subpopulations of *Ctrl+Hoxa9* transplanted marrow shown in Fig 3A.

Jalnapurkar et al, Figure S6

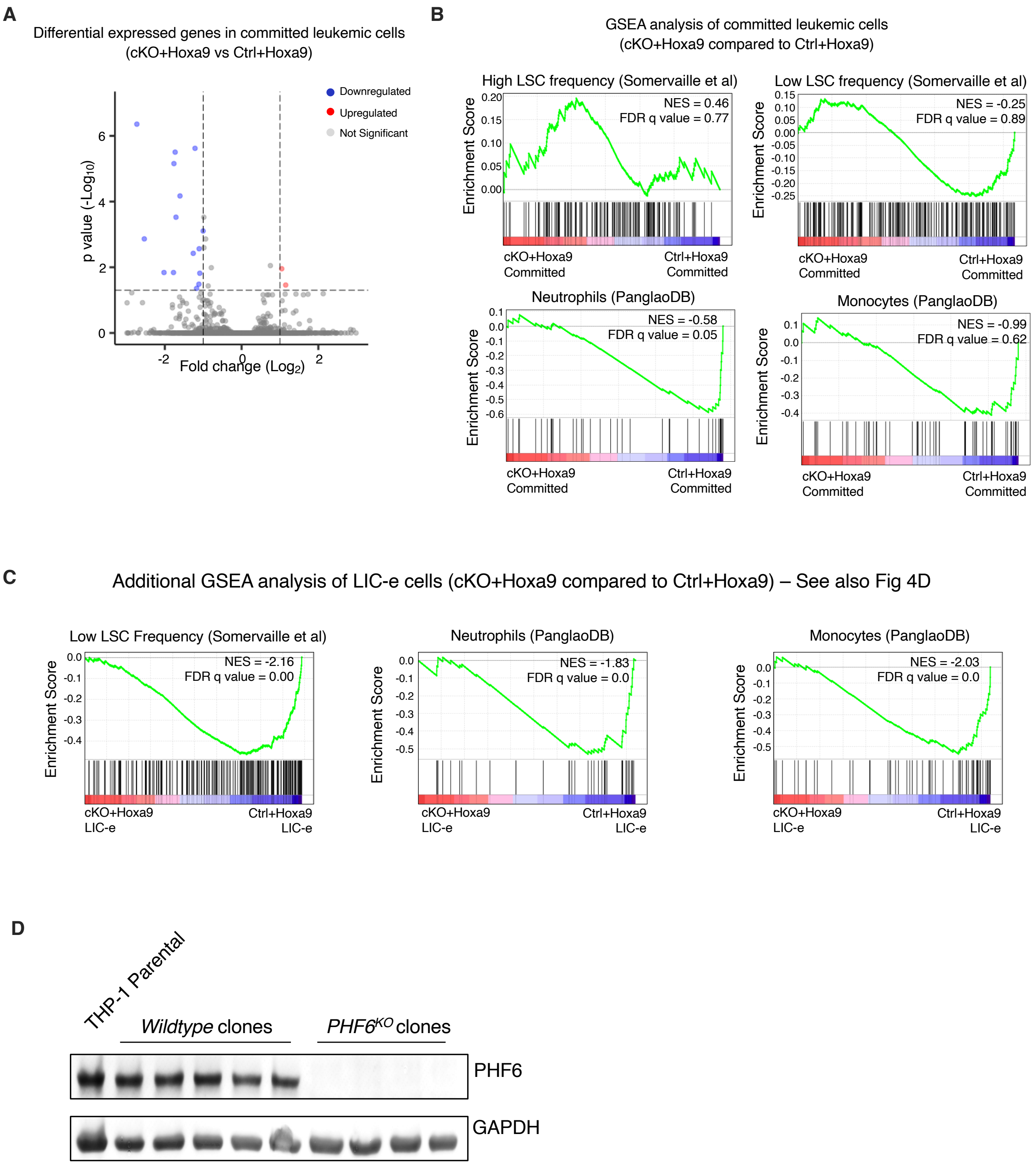

Figure S6 (related to Figure 4). ***Phf6*** **loss has minimal effects on the transcriptome of the committed (c-Kit+, Ly6C+) AML cell population**

A, Volcano plot showing differentially expressed genes between sorted committed cells (c-Kit+, Ly6C+) from *Ctrl+Hoxa9* and *cKO+Hoxa9* mice at 8 weeks after transplantation. Minimal gene expression changes were seen, with 28 downregulated and 3 upregulated genes in *cKO+Hoxa9* compared to *Ctrl+Hoxa9.*

B, Gene set enrichment analysis (GSEA) of the *cKO+Hoxa9* committed cell transcriptome compared to *Ctrl+Hoxa9* for gene sets related to high and low LSC frequency, mature neutrophils, and mature monocytes. Normalized Enrichment scores (NES) and FDR q values are shown.

C, Gene set enrichment analysis (GSEA) of the *cKO+Hoxa9* LIC-e transcriptome compared to *Ctrl+Hoxa9* for gene sets related to low LSC frequency, mature neutrophils, and mature monocytes. Normalized Enrichment scores (NES) and FDR q values are shown.

D, Immunoblot analysis verifying loss of PHF6 expression in human THP-1 $PHF6^{KO}$ clones compared to *wildtype* clones.

Jalnapurkar et al, Figure S7

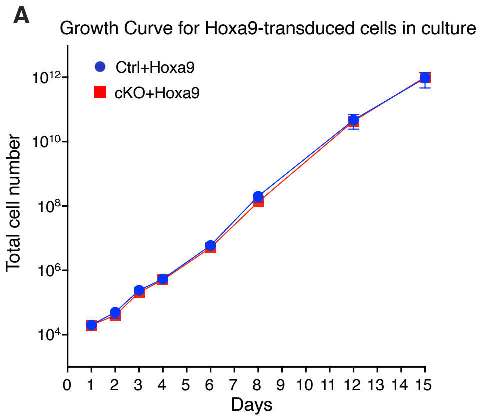

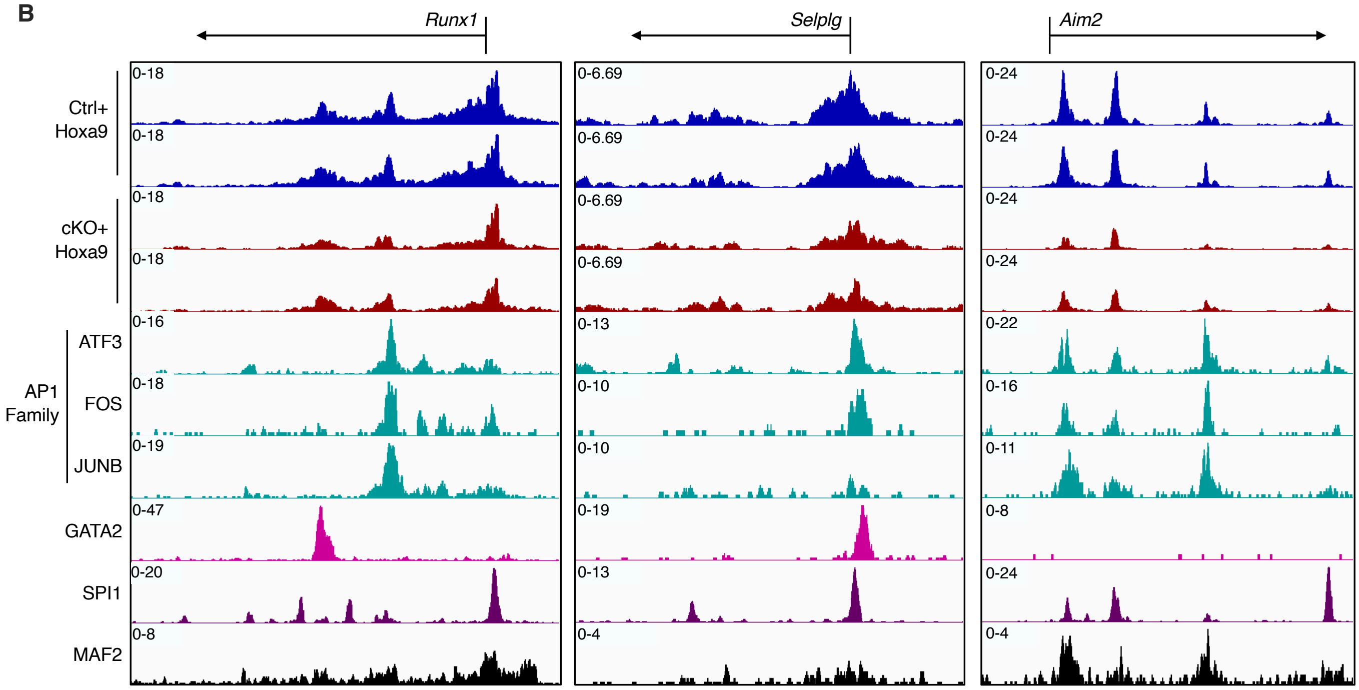

Figure S7 (related to Figure 6). Tracks of transcription factor occupancy at differentially expressed genes

A, Growth curve of *in vitro* cultured *Ctrl+Hoxa9* and *cKO+Hoxa9* cells.

B, IGV browser tracks of ATAC-Seq signal in LIC-e cells at promoters of genes downregulated or unchanged in *cKO+Hoxa9* along with ChIP-Seq signal of proteins whose motifs are enriched in regions of reduced accessibility in *cKO+Hoxa9* LIC-e *cells.*

Ctrl+Hoxa9 and *cKO+Hoxa9* peaks are scaled to the same Y-axis. Transcription factor ChIP-Seq tracks are taken from publicly available datasets in myeloid or leukemic cell types (see Table S3).
