# Supplementary material for: PHF6 suppresses self-renewal of leukemic stem cells in AML
Source: Leukemia. Author manuscript; Available in PMC 2024 Sep 1. (PMC11347380; doi:10.1038/s41375-024-02340-5)
Supplement: Supplemental Methods [file NIHMS2009447-supplement-Supplemental_Methods.pdf]

SUPPLEMENTAL MATERIAL AND METHODS

Analysis of BEAT AML data

Mutational and survival information for 783 human adults with AML was obtained from the BEAT AML[26] dataset (through the cBioPortal website https://www.cbioportal.org/, RRID:SCR_014555). Patients were stratified based on ELN2017 classification provided on cBioPortal. Kaplan-Meier survival curve was plotted for adverse ELN risk groups (*PHF6* mutations were rare in intermediate risk AML), and statistical significance was assessed using the Log-rank (Mantel- Cox) test. For Kaplan-Meier survival curves based on mutation type, all non-favorable patients with PHF6 mutations (22 patients) were considered.

For transcriptome analysis, 671 patient samples with available RNA-Seq data were considered and filtered to exclude ELN favorable patients (which do not co-occur with *PHF6* mutations). The resulting 502 patients were categorized according to *PHF6* mutated and unmutated status, and high *HOXA9* expression (log2 RNA-Seq RPKM>1). GSEA analysis was performed using DESeq2 normalized read counts using GSEAv4.1.0. Mac application (SeqGSEA, RRID:SCR_005724, Broad institute) between *PHF6*^{Mut} *HOXA9* high and *PHF6*^{WT} *HOXA9* high patient cohorts.

Cell Culture and generation of *PHF6*^{KO} and wildtype clones

THP-1 cells were a gift from Martin Carroll at the University of Pennsylvania. Cells were cultured in RPMI-1640 (Gibco, 11875085) supplemented with 10% FBS (GeminiBio, 100-106) and 1% PenStrep (Gibco, 15070063). Cell numbers were maintained between 0.3 to 1 million/ml for optimal growth conditions. HEK-293T cells were cultured in DMEM (Gibco, A4192101) supplemented with 10% FBS and 1% PenStrep. For generation of *PHF6*^{KO} and wildtype clones, four targeted guide RNA (gRNA) sequences against human PHF6 (at exons 2, 3, 4) and five control (non-targeting) gRNA sequences were designed using Zhang Lab gRNA design tools (https://zlab.bio/guide-design-resources) and cloned into lentiCRISPRv2 lentiviral plasmid expressing GFP (Addgene, 52961). For viral production, the ligated plasmid (4 µg) and

packaging plasmids (2 µg gag-pol (pCAG-kGP1-1R), 0.67 µg envelope (pHDM-G), and 0.67 µg rev/tat (pCAG-RTR2)) were mixed with JetPRIME transfection reagent and buffer (Polyplus, 89129-922) and added to HEK293T cells in a 10cm plate. Media was replaced with fresh media 24 hrs post-transfection, and viral supernatant was collected after 72 hrs and used to spin-infect THP-1 cells at 2400 RPM for 90 mins at 37°C with 8µg/ml of polybrene. Clones were grown after single -cell sorting for GFP expression, and one clone per gRNA was selected after confirmation of wildtype or knockout status using Sanger sequencing and immunoblot analysis.

Immunoblotting

Around 1 million cells were lysed in RIPA buffer containing protease inhibitors such as chymostatin, PMSF, PIC, a phosphatase inhibitor, and nuclease-free water. The protein concentrations were measured using Thermo Fisher's BCA assay kit. The proteins were separated by SDS-PAGE and transferred onto a PVDF membrane. The membrane was blocked using 5% milk and incubated with primary antibodies against PHF6 (Cat # sc-271767, Santa Cruz, CA, USA) and GAPDH (Cat # 2118L, Cell Signaling, MA, USA) at dilutions of 1:250 and 1:2000, respectively. Protein bands were visualized using the Odyssey CLx Imaging System (LI-COR Biosciences, NE, USA).

***Hoxa9* and *MLL-AF9* overexpression construct and retrovirus preparation**

pMSCV Hoxa9-IRES-GFP and *pMSCV MLL-AF9-IRES-GFP* retroviral construct plasmids was a generous gift from Kathrin Bernt (Division of Oncology, Children's Hospital of Philadelphia). Retroviruses were generated by transient co-transfection of 293T cells with retroviral vector and ecotropic packaging plasmids. Supernatant containing retroviruses was collected after 48 and 72 hrs of culture, filtered through a 0.45 µm filter, and used to transduce mouse cells.

***Hoxa9* and *MLL-AF9* transduction of mouse bone marrow**

Age-matched *Vav-Cre*$^{Cre/+}$ *Phf6*$^{+/Y}$ (*Ctrl*) or *Vav-Cre*$^{Cre/+}$ *Phf6*$^{fl/Y}$ (*cKO*) littermate mice were intraperitoneally injected with 150 mg/kg 5-fluorouracil (5-FU, Fresenius Kabi, 101720), and bone marrow cells were isolated from femurs after 4 days. Red blood cells were lysed with ACK buffer (Lonza, #10-548E), and cells were cultured for 2 days in IMDM (Thermo Fisher Scientific, #12440053) with 15% fetal bovine serum (Gemini Bio, #100-100), 20 ng/ml mSCF (Peprotech, #250-03), 20 ng/ml mTPO (Peprotech, #315-14), 20 ng/ml mFLT3 ligand (Peprotech, #250-31L), 10 ng/ml mIL3 (Peprotech, #213-13), and 10 ng/ml mIL6 (Peprotech, #216-16). Cultured cells were then transduced with spinofection (2400 RPM, 90 min) with *pMSCV Hoxa9-IRES-GFP* or *pMSCV MLL-AF9-IRES-GFP* retroviral supernatant and 4 ug/ml polybrene (Millipore Sigma, #TR-1003), and transferred to fresh media 18 hours after spin. GFP+ cells were purified after 2 days by fluorescence-activated cell sorting (BD FACSAria II).

mRNA isolation and qPCR

To confirm that *Phf6* cKO does not express *Phf6* mRNA, we isolated mRNA from bone marrow of 3 *Ctrl* and 3 *cKO* animals using TRizol (Thermo Scientific, #15596026) and prepared cDNA using 500 ng of total RNA using high capacity cDNA reverse transcriptase (Thermo Scientific, #4368813). qPCR for *Phf6* was carried out using primers TGTTTTCGTCTGCTTTGGTG (forward) and ATCATGCAATGCACAGTGGT (reverse), and for *Gapdh* (endogenous control) using primers CCCAGCTTAGGTTCATCAGG (forward) and GAATTTGCCGTGAGTGGAGT (reverse).

Immunophenotypic analysis and cell sorting

Total bone marrow was isolated by flushing and crushing bones (femur, tibia, humerus, ilium and column vertebralis) in PBS supplemented with 2% FCS. Red blood cell lysis was performed for 5 min at room temperature in ACK lysing buffer (Lonza, #10-548E). For flow cytometry, a single-cell suspension of bone marrow cells was stained with the requisite panel of fluorochrome -conjugated antibodies on ice for 30 minutes to 1 hour. A complete list of antibodies is provided in Table S1. Cells were analyzed using an LSR Fortessa flow cytometer (BD Biosciences), and data

were analyzed using FlowJo (FlowJo, LLC). When purification of cell populations was desired, cells were sorted using a BD Aria II sorter (BD Biosciences) into chilled PBS with 10% BSA for further processing.

Colony Forming assay

For myeloid clonogenic progenitor assays, *Hoxa9*-transduced or *MLL-AF9*-transduced cells were plated on 35 mm Petri dishes (Corning) in a 1.1 mL culture mixture containing Methocult (STEMCELL Technologies, #M3234) with 20 ng/ul mIL3 (Peprotech, #213-13), 20 ng/ul mIL6 (Peprotech, #216-16), and 20 ng/ul mSCF (Peprotech, #250-03). Colonies propagated in culture were scored and replated (500 cells/plate) every 7 days. Average colony size was determined by counting the number of cells obtained per plate and dividing by the number of colonies. A total of 8 replating rounds were performed.

***In vitro* culture of *Hoxa9*-transduced cells**

Hoxa9-transduced bone marrow cells from *Ctrl* or *cKO* mice were sorted for GFP positivity, and cultured in IMDM (Thermo Scientific, #12440053) with 15% fetal bovine serum (Gemini Bio), 20 ng/ml mSCF (Peprotech, #250-03), 20 ng/ml mTPO (Peprotech, #315-14), 20 ng/ml mFLT3 ligand (Peprotech, #250-31L), 10 ng/ml mIL3 (Peprotech, #213-13), and 10 ng/ml mIL6 (Peprotech, #216-16). Cells were diluted to 100K/ml every other day.

AML mouse model and serial bone marrow transplantation

For all transplantation experiments, recipient mice were wild-type C57BL/6J mice aged 8-20 weeks, lethally irradiated with 1100cGy administered as two split doses of 550cGy given 4 hours apart. *Hoxa9*-transduced bone marrow cells from *Ctrl* or *cKO* mice were injected retro-orbitally into primary recipient mice along with 400K radioprotectant cells of healthy age-matched bone marrow. Mice were monitored for activity and body conditioning score. For survival curve cohorts, mice were euthanized when humane endpoints for morbidity were met. For serial transplantation, bone marrow from primary recipient mice was harvested at 8 weeks after transplantation and

lysed with ACK buffer. Sorted GFP+ cells along with 400K radioprotectant cells were retro-orbitally injected into lethally irradiated secondary recipients. Subsequent tertiary transplants were similarly performed using sorted GFP+ cells from marrow harvested from secondary recipients at 8 weeks after transplantation. Each cohort contained 8-20 week old age and sex-matched recipient mice.

Limiting dilution transplantation assay

To calculate LIC (Leukemia initiating cell) frequency in freshly transduced marrow or in 8-week transplanted marrow, cohorts of lethally irradiated recipient mice were transplanted with 400K, 100K, 30K, 3K or 1K GFP+ cells from the respective source. Mice were euthanized when humane endpoints for morbidity were met or when >375 days had passed (experiment termination), and analyzed for the presence of GFP+ cells in bone marrow. All mice that achieved humane endpoints for morbidity had >80% GFP+ cells in marrow, and were counted as responders to the corresponding dose. At experiment termination (>375 days), a small number of mice with >1% GFP were also counted as responders (most had nearly 80% GFP positivity), while mice with <1% GFP+ cells in marrow and peripheral blood were counted as non-responders. LIC frequency was calculated using ELDA software [42].

Histopathology

Mouse femur, spleen, liver, and lungs were harvested and fixed in formaldehyde. Femurs were decalcified and tissue paraffin blocks and slides were prepared and stained by the University of Pennsylvania Molecular Pathology and Imaging Core. Bone marrow was cytospun and peripheral blood was smeared onto glass slides, following which the slides were air-dried and stained using HEMA 3 stain (Thermo Fisher Scientific, #22-122911). Slides were imaged using a Leica microscope.

***In vivo* cell cycle analysis**

The BD Pharmingen™ APC BrDU Kit (BD Biosciences, #552598) was used as per the manufacturer's protocol. 1 mg/mouse BrdU (BD bioscience, #552598) was administered

intraperitoneally at 8 weeks after primary transplant, and bone marrow was harvested after 2 hours. Following ACK lysis, cells were stained for surface antigens, fixed and treated with DNase to expose BrdU antigen, and incubated with Pacific Blue conjugated anti-BrdU antibody as per the instruction manual. Flow cytometry was performed as described above.

***In vitro* cell cycle analysis**

For cell cycle analysis and to calculate the length of the cell cycle, the Click-iT™ EdU Pacific Blue™ Flow Cytometry Assay kit (Invitrogen™, #C10418) was used as per the manufacturer's protocol. Briefly, 50K sorted LIC-e cells were incubated for 10 hours in media containing 10 µM EdU. Cells were harvested every hour, washed with 1% BSA in PBS, and stained for surface antigens. Cells were then fixed using the Click-iT fixative for 15 minutes at room temperature, and subsequent washes were performed using the 1X Click-iT saponin-based permeabilization and wash reagent. Cells were incubated for 30 minutes at room temperature in the dark with the Click-iT reaction cocktail composed of $CuSO_4$, Pacific Blue dye, reaction buffer additive and PBS, following the manufacturer's instructions. Flow cytometry was performed as described above.

For EdU pulse-chase experiments, 50K purified LIC-e cells were incubated for 2 hours in media containing 10 µM EdU (pulse). Cells were washed 3 times with fresh media to remove EdU, and were cultured for an additional 8 hours (chase) before being harvested and stained as described above.

Apoptosis

Apoptotic and necrotic cell analysis was performed using Pacific Blue™ Annexin V Apoptosis Detection Kit with 7-AAD (BioLegend, #640926) as per manufacturer's instructions. Cells were distinguished as live (AnnexinV-, 7AAD-), apoptotic (AnnexinV+, 7AAD-), necrotic (7AAD+).

RNA sequencing

50K LIC-e and 150K committed leukemic cells were sorted from bone marrow of primary recipients of *Ctrl+Hoxa9* and *cKO+Hoxa9* cells at 8 weeks after transplantation. Total RNA was extracted

using TRIzol LS reagent (Thermo Fisher Scientific, #10296010) and RNeasy Mini Kit (Qiagen, #74106). Total RNA concentration and RNA integrity number was determined using a Bioanalyzer (Agilent Technologies) and high sensitivity RNA Screentape (Agilent, #5067-5579). Samples with RNA integrity number 8 or higher were used for RNA library preparation. For LIC-e cells, RNA libraries were prepared using Smarter Stranded Total RNA-Seq Kit - Pico Input Mammalian (Takara Bio, #635005). For committed cells, RNA libraries were prepared using NEBNext Ultra RNA Library Prep Kit for Illumina (New England Biolabs, #E7530L). All libraries were prepared using manufacturer's instructions. Libraries were quantified using high sensitivity D1000 Screentape (Agilent Technologies, #5067-5584) and KAPA Library Quantification Kit (Thermo Fisher Scientific, #KK4824). Equimolar libraries from each sample were pooled and subjected to 75-bp paired-end sequencing on a NextSeq 550 machine (Illumina). Raw reads were demultiplexed and FASTQ files were generated using Bcl2fastq v2.20.0.422 (Illumina). The reads were trimmed for low quality/adapter sequence using Trimmomatic (RRID:SCR_011848) [43], followed by aligning to the mouse genome (mm10) with STAR v2.7.10b aligner [44]. Gene-level read counts were generated using the featureCounts tool from Rsubread v2.0.3) [45]. Read count normalization and differential gene expression testing was performed using DESeq2 v1.30.1 [46]. GSEA analysis was performed using DESeq2 normalized read counts using GSEAv4.1.0. Mac application (SeqGSEA, RRID:SCR_005724, Broad institute). Gene signatures were downloaded from the MSigDB (Broad) or from the GSE datasets directly in the GSEA application. Gene set enrichment data was represented using default parameters and an FDR cutoff of 0.05.

For THP-1 clones, 100K cells were used for each clone (PHF6 KO n=4, wildtype n=5). Total RNA was extracted using RNeasy Mini Kit (Qiagen, #74106) and quantified and prepared for sequencing as described above using NEBNext Ultra RNA Library Prep Kit. Libraries were quantified, denatured, sequenced and analyzed as described above.

ATAC sequencing

After 4 days of *Hoxa9* transduction, 50K LIC-e cells were sorted from *Ctrl+Hoxa9* and *cKO+Hoxa9* bone marrow cultures. Cells were lysed and their nuclei were isolated, followed by tagmentation performed using Nextera Tn5 transposase (Illumina Nextera ATAC seq kit, 20034197). Libraries were prepared using dual-end indexing using NEBNext Ultra II DNA Library Prep Kit (New England Biolabs, #E7645L) using the manufacturer's protocol. The size and concentration of each sample's library were determined using Agilent 2200 Tapestation and KAPA library quantification kit (Roche, #KK4824) respectively. Libraries from all samples were pooled in equimolar concentrations and denatured along with 1% of PhiX for paired-end sequencing. The pooled library was quantified using KAPA library quantification kit (Roche KK4824) and equimolar libraries were pooled and subjected to 75-bp paired-end sequencing on a NextSeq 550 machine (Illumina). Raw reads were demultiplexed and FASTQ files were generated. Reads were trimmed of contaminating adapter sequences using Trimmomatic [43] and aligned to the mouse genome (mm10) using Bowtie2 (RRID:SCR_016368) [47]. Peaks of transposase-accessible chromatin were called and quantified using MACS2 v2.2.7.1 [48]. Differential and consensus peak analyses were performed using the DiffBind v3.0.15 package in R software (RRID:SCR_012918). Motif analysis was also performed for all regions with significant differential accessibility after filtering out low- confidence regions using HOMER [49]. Public ChIP-Seq datasets (Table S4) for ATF3, FOS, JUN, GATA2, SPI1, MAF2, IRF4, IRF8, RELA and RELB were mapped to mm10 genome and used for generating metagene plots using SeqPlot software.

Statistical Analysis

Statistical analysis was performed using GraphPad Prism v9.5.1 (GraphPad Software). We assumed equal distribution of variance between groups and performed either 1-way Anova with Sidak's multiple comparison testing or Student's *t-test*. Kaplan-Meier curves were used to represent survival, where significance was calculated with the log-rank (Mantel-Cox) test. We considered results $p < 0.05$ as statistically significant and represented using $*p < 0.05$, $**p < 0.01$,

***p < 0.001; ****p < 0.0001. We analyzed serial limited dilution leukemia-initiating cell data using the ELDA tool[42].

Blinding

All histopathological analyses were performed in a blinded fashion.

Replication

All transplant experiments were performed in multiple cohorts of recipients, and data were compiled for final analyses.

Power Analysis

Power analyses for mouse experiments were at 90% confidence, significance level of 0.05.

Inclusion and Exclusion Criteria

N/A, no clinical trial data in this study

Attrition

N/A, no clinical trial data in this study

ADDITIONAL SUPPLEMENTARY REFERENCES

42 Hu Y, Smyth GK. ELDA: extreme limiting dilution analysis for comparing depleted and enriched populations in stem cell and other assays. *J Immunol Methods* 2009; **347**: 70–78.

43 Bolger AM, Lohse M, Usadel B. Trimmomatic: a flexible trimmer for Illumina sequence data. *Bioinformatics* 2014; **30**: 2114–2120.

44 Dobin A, Davis CA, Schlesinger F, Drenkow J, Zaleski C, Jha S *et al.* STAR: ultrafast universal RNA-seq aligner. *Bioinformatics* 2013; **29**: 15–21.

45 Liao Y, Smyth GK, Shi W. featureCounts: an efficient general purpose program for assigning sequence reads to genomic features. *Bioinformatics* 2014; **30**: 923–930.

46 Love MI, Huber W, Anders S. Moderated estimation of fold change and dispersion for RNA-seq data with DESeq2. *Genome Biol* 2014; **15**: 550.

47 Langmead B, Salzberg SL. Fast gapped-read alignment with Bowtie 2. *Nat Methods* 2012; **9**: 357–359.

48 Feng J, Liu T, Qin B, Zhang Y, Liu XS. Identifying ChIP-seq enrichment using MACS. *Nat Protoc* 2012; **7**: 1728–1740.

49 Ginhoux F, Guilliams M, Naik S. *Dendritic Cell and Macrophage Nomenclature and Classification*. Frontiers Media SA, 2016.
